# Progesterone receptor membrane component 1 (PGRMC1) binds and stabilizes cytochromes P450 through a heme-independent mechanism

**DOI:** 10.1016/j.jbc.2021.101316

**Published:** 2021-10-20

**Authors:** Meredith R. McGuire, Debaditya Mukhopadhyay, Stephanie L. Myers, Eric P. Mosher, Rita T. Brookheart, Kai Kammers, Alfica Sehgal, Ebru S. Selen, Michael J. Wolfgang, Namandjé N. Bumpus, Peter J. Espenshade

**Affiliations:** 1 Department of Cell Biology, Johns Hopkins University School of Medicine, Baltimore, Maryland, USA; 2 Department of Pharmacology and Molecular Sciences, Johns Hopkins University School of Medicine, Baltimore, Maryland, USA; 3 Department of Oncology, Johns Hopkins University School of Medicine, Baltimore, Maryland, USA; 4 Department of Biological Chemistry, Johns Hopkins University School of Medicine, Baltimore, Maryland, USA

**Keywords:** cytochrome P450, heme, enzyme degradation, drug metabolism, liver metabolism, protein turnover, ALT, alanine aminotransferase, AST, aspartate aminotransferase, ECOD, 7-ethoxycoumarin O-deethylation, PGRMC1, progesterone receptor membrane component 1

## Abstract

Progesterone receptor membrane component 1 (PGRMC1) is a heme-binding protein implicated in a wide range of cellular functions. We previously showed that PGRMC1 binds to cytochromes P450 in yeast and mammalian cells and supports their activity. Recently, the paralog PGRMC2 was shown to function as a heme chaperone. The extent of PGRMC1 function in cytochrome P450 biology and whether PGRMC1 is also a heme chaperone are unknown. Here, we examined the function of Pgrmc1 in mouse liver using a knockout model and found that Pgrmc1 binds and stabilizes a broad range of cytochromes P450 in a heme-independent manner. Proteomic and transcriptomic studies demonstrated that Pgrmc1 binds more than 13 cytochromes P450 and supports maintenance of cytochrome P450 protein levels posttranscriptionally. *In vitro* assays confirmed that *Pgrmc1* KO livers exhibit reduced cytochrome P450 activity consistent with reduced enzyme levels. Mechanistic studies in cultured cells demonstrated that PGRMC1 stabilizes cytochromes P450 and that binding and stabilization do not require PGRMC1 binding to heme. Importantly, Pgrmc1-dependent stabilization of cytochromes P450 is physiologically relevant, as *Pgrmc1* deletion protected mice from acetaminophen-induced liver injury. Finally, evaluation of Y113F mutant Pgrmc1, which lacks the axial heme iron-coordinating hydroxyl group, revealed that proper iron coordination is not required for heme binding, but is required for binding to ferrochelatase, the final enzyme in heme biosynthesis. PGRMC1 was recently identified as the causative mutation in X-linked isolated pediatric cataract formation. Together, these results demonstrate a heme-independent function for PGRMC1 in cytochrome P450 stability that may underlie clinical phenotypes.

Cytochromes P450 are an essential superfamily of heme-containing monooxygenase enzymes that catalyze biosynthetic reactions, detoxify xenobiotic compounds, and metabolize pharmaceutical drugs (1). These enzymes have a characteristic catalytic cycle that allows for the safe activation of molecular oxygen to react with substrates (1). Cytochromes P450 are found in all kingdoms of life (1). In mammals, the liver is the primary site of expression (1, 2). Cytochromes P450 rely on protein–protein interactions between a cytochrome P450 and cytochrome P450 oxidoreductase and, in some cases, cytochrome b5 (CYB5) to transfer electrons to the cytochromes P450 to complete catalysis (1). Regulation of cytochrome P450 activity is crucial to maintain homeostasis and metabolize xenobiotics (1). Although the transcriptional regulation of cytochrome P450 activity is well-studied, the posttranslational control of cytochrome P450 activity is yet to be fully understood (1).

Progesterone receptor membrane component 1 (PGRMC1) participates in protein–protein interactions with cytochromes P450 in the fission yeast *Schizosaccharomyces pombe* and human cells (3). PGRMC1 affects cytochrome P450 activity in these systems by supporting the enzymatic activity of cytochrome P450 51A1 (CYP51A1) in cholesterol synthesis (3). In addition to supporting cytochrome P450 activity, PGRMC1 has been implicated in a wide variety of cellular processes (4, 5, 6, 7, 8, 9, 10, 11, 12, 13). In the female reproductive tract, PGRMC1 promotes fertility and plays an antiapoptotic role in the ovary (4, 5). In cancer, PGRMC1 conveys better growth and chemoresistance (6, 7, 8). In protein trafficking, PGRMC1 binds epidermal growth factor receptor (EGFR) and participates in EGFR trafficking to the plasma membrane (9). Additional processes that may involve PGRMC1 include glucose-stimulated insulin release from beta cells, autophagy, systemic iron homeostasis, and amyloid beta accumulation in neurons (10, 11, 12, 13). Given the large number of human and mouse cytochromes P450 (57 and 102, respectively), this broad range of reported functions for PGRMC1 is consistent with a common role in cytochrome P450 biology (14). However, the extent of PGRMC1-cytochrome P450 binding in mammals and the mechanism by which PGRMC1 affects cytochrome P450 activity are unknown.

PGRMC1 is a heme-binding membrane protein, and despite its name, PGRMC1 binding to heme is better understood than its binding to progesterone (15). As a type I transmembrane protein, PGRMC1 binds heme in a cytoplasmic CYB5-like domain (16). While this domain is structurally similar to CYB5, the characteristics of heme binding are distinctly different. CYB5 is an electron carrier that binds heme in a hexacoordinate fashion with histidine residues coordinating the heme iron molecule (17). PGRMC1 binds heme in a pentacoordinate fashion with the hydroxyl group of a tyrosine residue coordinating the iron in the heme molecule, making it unlikely that PGRMC1 is an electron carrier (18). The significance of PGRMC1 heme binding remains to be elucidated.

PGRMC1 has a paralog in mammals, named progesterone receptor membrane component 2 (PGRMC2), with which it shares 60% amino acid identity. In a landmark study, the Saez lab demonstrated that PGRMC2 functions as a heme chaperone and plays a critical role in mitochondrial homeostasis in mouse brown adipose tissue (19). Heme chaperones are necessary because of the reactive nature of free heme. They sequester heme when it is taken up from the environment or synthesized in the mitochondria. PGRMC2 is required for delivery of newly synthesized heme from the mitochondria to the nucleus (19). These observations suggest that in addition to its demonstrated role in cytochrome P450 biology, PGRMC1 may also function as a heme chaperone. PGRMC1 and PGRMC2 have overlapping but different subcellular localizations. Both proteins are found in the endoplasmic reticulum (16, 20), where cytochromes P450 reside. PGRMC2 is found in the nucleus where it delivers heme to the nuclear receptor Rev-Erb (19). PGRMC1 has also been reported to localize to the nucleus, mitochondria, and plasma membrane (5, 19, 21). Here, we examined the function of PGRMC1 in mouse liver using a knockout model and found that PGRMC1 binds and stabilizes a broad range of cytochromes P450 in a heme-independent manner, defining a nonheme chaperone function for this family of proteins.

## Results

### Generation of a Pgrmc1 KO mouse and liver characterization

To study the function of PGRMC1 *in vivo*, we generated mice with a conditionally targeted *Pgrmc1* allele. The conditional allele contains loxP sites flanking exons 1 and 2 of *Pgrmc1* with a neomycin resistance cassette (Fig. S1*A*). Mice carrying the conditionally targeted allele were crossed to mice expressing *Sox2*-Cre recombinase to produce whole body *Pgrmc1* knockout (KO) mice. Male and female whole body *Pgrmc1* KO mice are viable. Because *Pgrmc1* is X-linked (22) and cytochrome P450 expression is known to be sexually dimorphic (23), only male *Pgrmc1* KO mice were examined in this study. A complete blood count and full clinical chemistry were performed on *Pgrmc1* KO mice (Tables S1.1 and S1.2). While some hematology parameters (red blood cell count, hematocrit, and hemoglobin) were 10% lower in the KO mice, the mice were not anemic as the values were within reference range (24, 25). Similarly, the KO mice had 8% less plasma cholesterol than wild-type (WT) mice. This value indicates that the KO mice had a greater challenge maintaining systemic cholesterol homeostasis, but this was a subclinical phenotype (24). In summary, *Pgrmc1* KO mice were in good health both clinically and physically.

**Figure 1:**
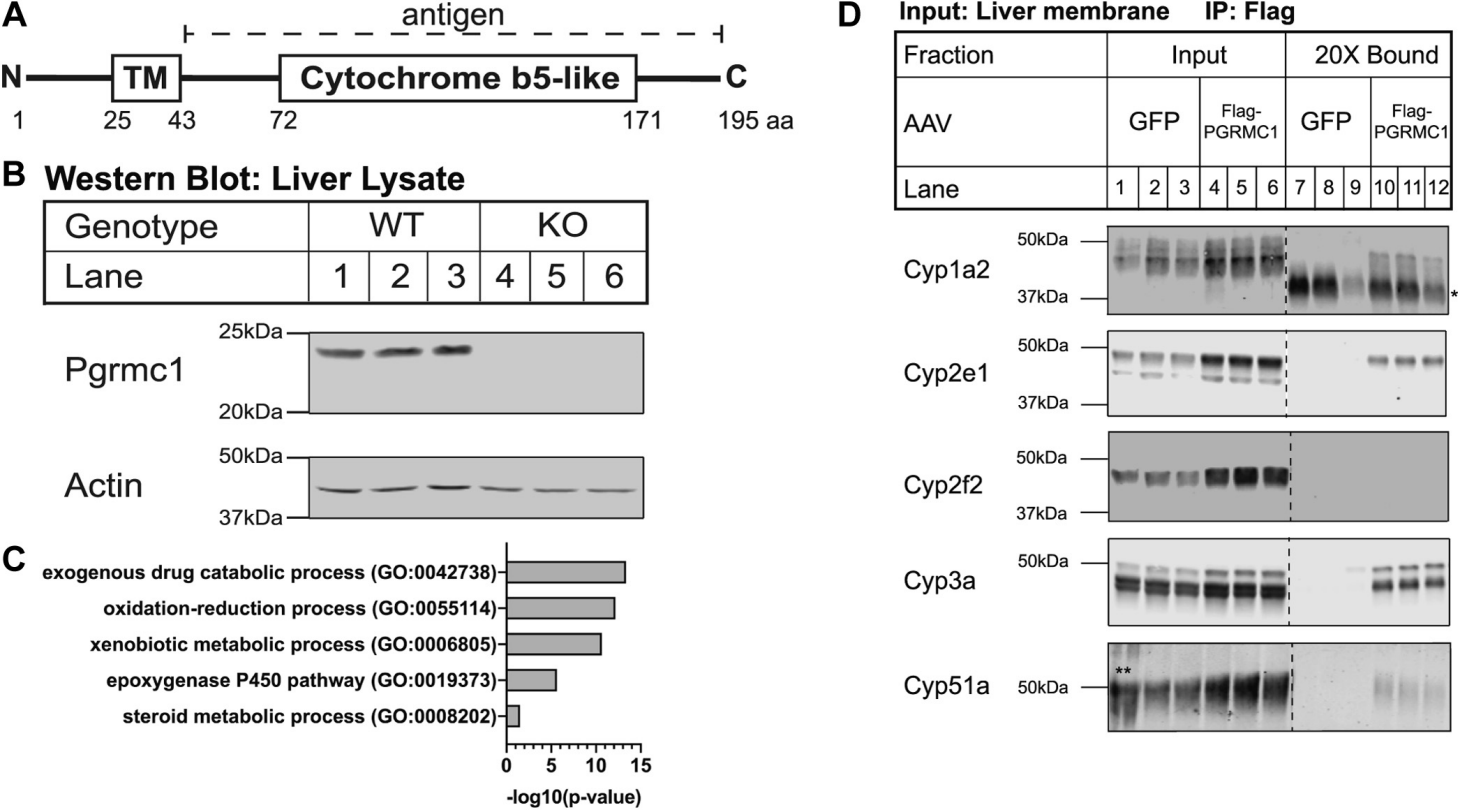
**Pgrmc1 binds cytochromes P450 in mouse liver.***A*, schematic of human PGRMC1 protein (Uniprot O00264). The 195 amino acid protein consists of a single-pass transmembrane domain (TM) and cytochrome b5-like domain, which shares 30% identity with the human cytochrome b5 protein (NP_683725.1). A rabbit polyclonal antibody (5944) was raised to a bacterially expressed recombinant protein consisting of amino acids 43 to 195 of human PGRMC1. *B*, Pgrmc1 protein expression in *Pgrmc1* KO mouse liver. *Pgrmc1* was knocked out in the whole animal by crossing mice with a conditionally targeted *Pgrmc1* allele containing loxP sites flanking exons 1 and 2 of the gene to *Sox2-Cre* mice. Knockout in the liver was confirmed by western blotting liver lysate (+β-mercaptoethanol) with an anti-PGRMC1 antibody (5944). Actin is a loading control. Each lane is a biological replicate (WT n = 3, KO n = 3). *C*, biological process gene ontology (GO) Term analysis on candidate binding partners of Flag-Pgrmc1 from liver. *Pgrmc1* KO mice were infected with 5 × 10^11^ particles of AAV8 GFP or AAV8 Flag-Pgrmc1 by tail vein injection and sacrificed after 8 days. Liver membrane fractions were subjected to Flag coimmunoprecipitation. Eluates from technical triplicates were pooled for each of three biological replicates and tagged with isobaric labels. Flag-Pgrmc1-binding proteins were identified by mass spectrometry—33 proteins have a fold-change ≥20% compared with the GFP control. The GO terms enriched in relation to the complete *Mus musculus* proteome were identified using PANTHER. A Fisher’s exact test and Bonferroni correction were used to determine enriched GO terms with a *p*-value ≤ 0.05. *D*, input (×) and bound (20×) fractions from Flag coimmunoprecipitation samples were subjected to western blotting for cytochromes P450 detected by mass spectrometry. Each panel is a montage from a single membrane with *dashed lines* denoting removed lanes. (∗ denotes IgG; ∗∗ ladder overflow into Lane 1.)

Both PGRMC1 and cytochromes P450 are highly expressed in human and murine liver, so we focused our study on this tissue. Knockout of Pgrmc1 protein was confirmed in *Pgrmc1* KO mouse liver by western blotting using an anti-PGRMC1 antibody raised against human PGRMC1 (amino acids 43–195) (Fig. 1, *A* and *B*) (1, 3). Liver size and liver tissue histology were both normal (Fig. S1, *B* and *C*) (26), and plasma alanine aminotransferase (ALT) level, a marker of liver injury, was not elevated (Table S1.2). Liver metabolite amounts assayed were similar to control values (Table S1.3), except for a small decrease in glutamine (9%). Overall, the livers of *Pgrmc1* KO mice were healthy.

### Pgrmc1 binds cytochromes P450 in the liver

Our previous studies demonstrated that PGRMC1 binds cytochromes P450 in yeast and human cells (3). To test whether Pgrmc1 binds cytochromes P450 in mouse liver, *Pgrmc1* KO mice were infected with AAV8 *GFP* or AAV8 *Flag-Pgrmc1* to express the protein in the liver for 8 days (Fig. S2, *A* and *B*). A Flag affinity purification was performed on detergent-solubilized liver membrane fractions from these mice (Fig. S2*C*), and bound proteins were identified by mass spectrometry. Flag-Pgrmc1 binding partners (Tables S2.1 and S2.2) included 32 proteins, of which 13 (41%) were cytochromes P450. In fact, the most enriched gene ontology (GO) term among the candidate binding partners was “exogenous drug catabolic process” (*p* = 4.35 × 10^−14^), which reflects the cytochromes P450 (Fig. 1*C*). The second most enriched GO term was “oxidation-reduction process” (*p* = 6.70 × 10^−13^), which reflects the cytochromes P450 and additional proteins involved in electron transfer reactions, such as lathosterol oxidase and retinol dehydrogenase. Binding of Flag-Pgrmc1 to Cyp1a2, Cyp2e1, Cyp3a, and Cyp51a in the liver membrane fraction was validated by western blotting; binding to Cyp2f2 was not confirmed (Fig. 1*D*). In each confirmed instance, Flag-Pgrmc1 bound to 0.2% to 2% of cytochrome P450 protein in the liver membrane fraction (Fig. S2*D*). This coimmunoprecipitation experiment demonstrated that Flag-Pgrmc1 binds cytochromes P450 and, more broadly, may bind enzymes involved in redox processes in mouse liver.

### Pgrmc1 functions to maintain cytochrome P450 protein levels posttranscriptionally

**Figure 2:**
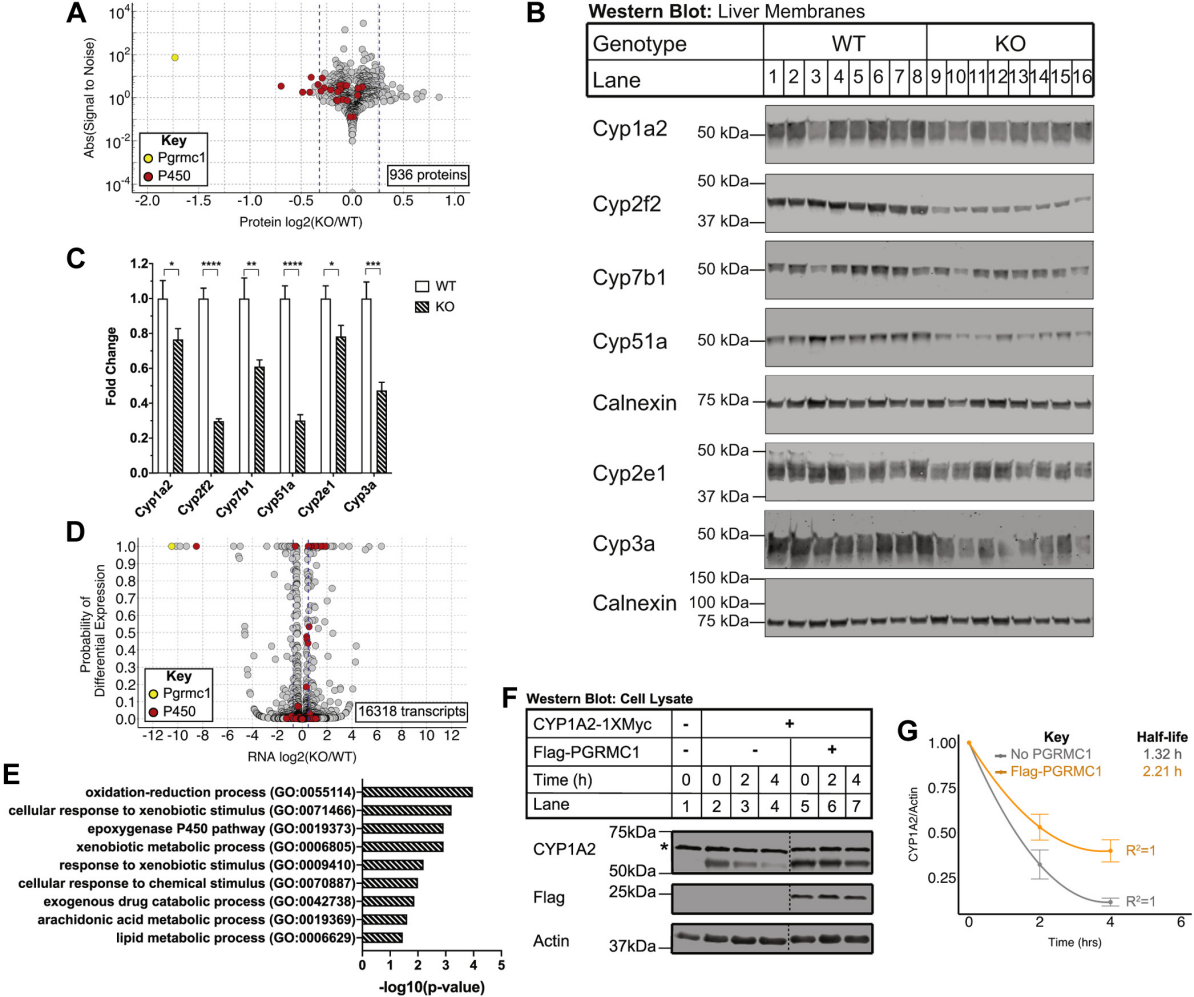
**Pgrmc1 regulates cytochrome P450 protein levels.***A*, membrane-enriched proteome of *Pgrmc1* KO livers. Steady-state protein levels from WT and *Pgrmc1* KO liver membrane fractions were quantified by mass spectrometry with isobaric tagging. Membrane proteins from the livers of four to five male mice of each genotype were pooled. Two biological replicates were conducted with 936 proteins measured in both replicates and further analyzed. The log2 fold-change [log2(KO/WT)] in expression was plotted against the absolute value of the signal-to-noise ratio. The signal-to-noise ratio is a moderated test statistic and reflects how unusually a given value of the log2 fold-change is when considering the whole data set. Proteins with an absolute value of the signal-to-noise ratio ≥2 were considered significant. *Blue dashed lines* indicate a 20% fold change, cytochromes P450 are colored *red*, and Pgrmc1 is colored *yellow*. *B*, Western blots of liver membrane fractions for cytochromes P450. Liver membrane-enriched protein (15 μg/lane for Cyp2f2, Cyp51a, and Cyp7b1, 10 μg/lane for all others; +β-mercaptoethanol; calnexin panels are loading controls for the panels above them) was analyzed by western blotting using the indicated antibodies. Each lane is a biological replicate (WT n = 8, KO n = 8). *C*, fold change in liver membrane protein expression of cytochromes P450 in *Pgrmc1* KO compared with WT mice for (*B*). Cytochrome P450 signal intensities for each lane in (*B*) were first normalized to calnexin. Error bars are 1 SEM. (WT n = 8, KO n = 8; Welch’s *t* test, one-tailed; ∗ *p* ≤ 0.05, ∗∗ *p* ≤ 0.01, ∗∗∗ *p* ≤ 0.001, ∗∗∗∗ *p* ≤ 0.0001). *D*, transcriptome of *Pgrmc1* KO livers. RNA-seq was performed on total RNA from *Pgrmc1* KO mice and WT controls. RNAs from five mice per genotype were pooled to produce one sample per genotype for analysis. In total, 16,318 genes were measured and plotted. The log2 fold change [log2(KO/WT)] in expression was plotted against the probability of differential expression (PDE), which is the Bayesian posterior probability that a difference in expression exists. Genes with a PDE ≥0.95 were considered significant. *Blue dashed lines* indicate a 40% fold change, cytochromes P450 are colored *red*, and Pgrmc1 is colored *yellow*. *E*, biological process GO Term analysis on transcripts more abundant in *Pgrmc1* KO liver. Enriched GO terms among the 70 genes with a fold-change ≥40% and PDE ≥0.95 as compared with all transcripts measured were identified using PANTHER. A Fisher’s exact test and Bonferroni correction were used to determine enriched GO terms with a *p*-value ≤ 0.05. *F*, flag-PGRMC1 stabilizes CYP1A2 in human SV589 cells. *PGRMC1* KO cells were cotransfected with 5 μg CYP1A2-1XMyc and 10 μg of empty vector (pcDNA3.1) or Flag-PGRMC1 in a 10-cm plate. At 24 h posttransfection, cells were split 1:6 into a 6-well plate. At 48 h posttransfection, cells were treated with 100 μg/ml emetine and harvested every 2 h. Cell lysates were analyzed by western blotting. Actin is a loading control. Panels are representative of five independent experiments. Each panel is a montage from a single membrane with *dashed lines* denoting removed lanes. (∗ denotes background band). *G*, the half-life of CYP1A2 in the presence and absence of Flag-PGRMC1 was determined from (*F*). CYP1A2 signal was normalized to the actin loading control signal. Within each replicate, expression was normalized to the t = 0 value for each condition and then averaged. The data were fit to a second-order polynomial linear model (R^2^ WT = 1, KO = 1). The half-life is calculated as the x-coordinate of the curve when y = 0.5. Error bars are 1 SEM (No PGRMC1 n = 5, Flag-PGRMC1 n = 5).

We consistently noted an increase in levels of cytochromes P450 expression in Flag-Pgrmc1-expressing KO liver as compared with GFP-expressing KO liver (Figs. 1*D* and S2*E*). To determine if Pgrmc1 affects cytochrome P450 protein levels globally, we performed quantitative mass-spectrometry proteomics with isobaric tagging on liver membrane samples from WT and *Pgrmc1* KO mice and detected a total of 936 proteins in both of two biological replicates (Tables S3.1--S3.6). We detected 23 cytochromes P450 (Fig. 2*A*, Table S3.1), a comparable number to a previous proteomic study that surveyed cytochromes P450 in mouse liver (27). Differentially expressed proteins were defined as those increased or decreased by at least 20% and a signal-to-noise ratio of at least 2 (Tables S3.2 and S3.3). Five core proteasomal subunits were more abundant (20–26% increase) in *Pgrmc1* KO samples. Pgrmc1 was the least abundant protein (70% decrease), with its detection in the *Pgrmc1* KO samples likely an artifact of coisolation interference during the mass spectrometry run (28). Among the other four decreased proteins (21–38%) in *Pgrmc1* KO samples were three cytochromes P450, including Cyp2f2, Cyp7b1, and Cyp3a13. While not all detected cytochromes P450 met the cutoff criteria for significant change and signal-to-noise ratio, the P450 family tended to be decreased in *Pgrmc1* KO liver (Fig. 2*A*). Protein expression of Cyp1a2, Cyp2f2, Cyp7b1, Cyp51a, Cyp2e1, and Cyp3a was confirmed to be decreased in *Pgrmc1* KO mouse liver membranes by western blotting (Fig. 2, *B* and *C*). Quantitative proteomics revealed a 14 to 38% reduction in protein across these specific cytochromes P450, and western blotting revealed a 22 to 70% reduction. Together, these complementary methods show that Pgrmc1 functions to maintain protein levels of these cytochromes P450.

Although Pgrmc1 binds to cytochromes P450, Pgrmc1 may also affect protein levels indirectly by reducing cytochrome P450 transcript levels in the liver*.* To investigate this, we performed RNA-seq on WT and *Pgrmc1* KO liver. Expression of 16,318 genes was measured, including 83 cytochromes P450 (Fig. 2*D*, Tables S4.1–S4.3). mRNAs more abundant in *Pgrmc1* KO livers were also enriched for the GO terms, “exogenous drug catabolic process” (*p* = 2.46 × 10^−3^) and “oxidation-reduction process” (*p* = 2.07 × 10^−3^) (Fig. 2*E*). Notably, Pgrmc1 binding partners were enriched for these same GO terms (Fig. 1*C*). For the subset of the 83 cytochromes P450 detected in the RNA-seq data (Table S4.4), 15% were more abundant in *Pgrmc1* KO samples compared with wild-type, and 83% did not change. These data indicate that loss of Pgrmc1 does not reduce cytochrome P450 transcript amounts. In fact, loss of Pgrmc1 leads to the upregulation of transcripts involved in drug metabolism and redox processes, indicating that Pgrmc1 does not reduce cytochromes P450 levels through transcriptional regulation.

Our *in vivo* data indicate that *Pgrmc1* regulates cytochrome P450 protein levels through some posttranscriptional mechanism. We next sought to establish whether *Pgrmc1* regulates cytochromes P450 protein levels by altering protein stability. We chose CYP1A2 because it bound Flag-Pgrmc1 *in vivo* and is a constitutively expressed cytochrome P450 that metabolizes pharmaceutical drugs and other xenobiotics (29). We generated human *PGRMC1* KO cells by CRISPR-editing SV589 fibroblasts (30). Knockout of *PGRMC1* was validated by sequencing and western blotting (Fig. S3*A*). When the *PGRMC1* KO cells were transfected with Flag-PGRMC1, overexpressed PGRMC1 was twofold higher than endogenous PGRMC1 (Fig. S3*B*). Next, we sought to validate the PGRMC1-CYP1A2 binding observed in mouse liver using human cells. Specific binding between Flag-PGRMC1 and CYP1A2-1XMyc was confirmed by Flag coimmunoprecipitation in *PGRMC1* KO cells (Fig. S3*C*). To test the effect of PGRMC1 on CYP1A2 stability, *PGRMC1* KO cells were transfected with CYP1A2-1XMyc with or without Flag-PGRMC1. At 48 h posttransfection, cells were exposed to the irreversible translation inhibitor emetine, and protein levels were measured over time. PGRMC1 stabilized CYP1A2-1XMyc protein, increasing its half-life by 67% (Fig. 2, *F* and *G*). This experiment demonstrated that PGRMC1 stabilizes CYP1A2 posttranslationally.

### Pgrmc1 is required for maximal Cyp1a2 and Cyp2e1 activity in the liver

As Pgrmc1 supports protein levels of cytochromes P450, we wondered whether cytochrome P450 activity was reduced in *Pgrmc1* KO mice. Our previous work showed that PGRMC1 binds CYP51A1 and supports its activity in cholesterol synthesis in HEK293 cells (3). To directly measure cytochrome P450 enzyme activity in *Pgrmc1* KO liver, we assayed 7-ethoxycoumarin O-deethylation (ECOD) using membrane samples from WT and *Pgrmc1* KO mice. ECOD is mediated by CYP1A1 and CYP1A2 in humans and rodents with additional cytochromes P450 contributing to metabolism in humans (31). CYP1A1 expression is induced in response to the presence of its substrates, which include highly toxic polyarylhydrocarbons that laboratory animals are unlikely to encounter (32). Thus, effects of Pgrmc1 on ECOD activity in mouse liver likely report on Cyp1a2 activity.

**Figure 3:**
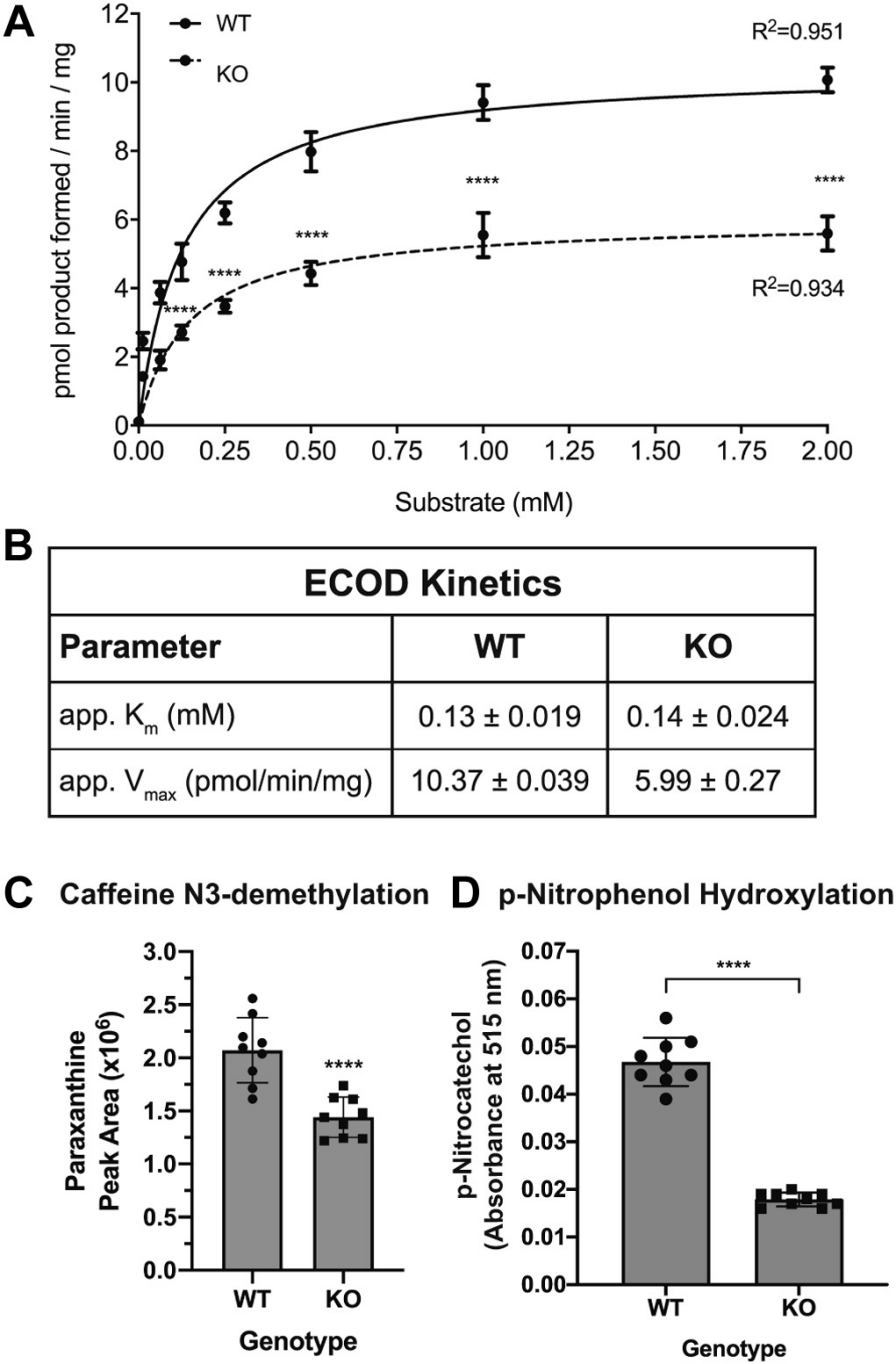
***Pgrmc1* KO mouse livers display reduced Cyp1a2 and Cyp2e1 activities.***A*, 7-ethoxycoumarin O-deethylation (ECOD) reaction in *Pgrmc1* KO membrane fractions. Increasing concentrations of 7-ethoxycoumarin (0, 0.0625, 0.125, 0.25, 0.5, 1, 2 mM) were combined with 40 μg of membrane protein from WT or *Pgrmc1* KO livers and the NADPH-dependent formation of 7-hydroxycoumarin in 30 min at 37 °C was assayed. Samples were pooled liver membrane protein from five male mice of each genotype. Enzyme kinetics of the ECOD reaction were fit to the Michaelis–Menten equation. Error bars are 1 SD (WT n = 3, KO n = 3 technical replicates; Student’s *t* test for each substrate concentration; ∗∗∗∗ *p* < 0.0001; nonlinear regression R^2^ WT= 0.951, KO= 0.934). *B*, apparent *K*_m_ and *V*_max_ of the ECOD reaction were calculated from the fitted Michaelis–Menten curves in (*A*). Values are mean ± SEM. *C*, caffeine N3-demethylation reaction in *Pgrmc1* KO membrane fraction. Liver membrane protein (100 μg) from WT or *Pgrmc1* KO mice prepared as in A was combined with 50 μM caffeine and an NADPH regeneration buffer system for 60 min at 37 °C. Paraxanthine formed was detected by mass spectrometry. Error bars are 1 SD (WT n = 9, KO n = 9 technical replicates; Student’s *t* test; ∗∗∗∗ *p* < 0.0001). *D*, p-Nitrophenol hydroxylation reaction in *Pgrmc1* KO membrane fraction. Samples were pooled liver membrane protein from four male mice of each genotype. Liver membrane protein (125 μg) from WT or *Pgrmc1* KO mice was combined with 100 μM p-nitrophenol and an NADPH regeneration buffer system for 60 min at 37 °C. Control samples contained no liver membrane protein. p-Nitrocatechol formed was detected spectrophotometrically and absorbances were corrected for background signal. Error bars are 1 SD (Control n = 6, WT n = 9, KO n = 9 replicates; Welch’s *t* test; ∗∗∗∗ *p* < 0.0001).

ECOD product formation (7-hydroxycoumarin) was monitored over a range of substrate (7-ethoxycoumarin) concentrations, and the data exhibited Michaelis–Menten kinetics (WT R^2^ = 0.951, KO R^2^ = 0.934) (Fig. 3*A*). Product formation was reduced at all substrate concentrations tested in the *Pgrmc1* KO samples, and the difference was statistically significant between 0.125 mM and 2 mM. The *K*_m_ of the reaction did not differ in the KO (Fig. 3*B*). However, the *V*_max_ of the reaction was 42% lower in the KO (Fig. 3*B*), consistent with reduced levels of Cyp1a2 protein in Pgrmc1 KO livers (Fig. 2, *B* and *C*).

Caffeine is another well-established Cyp1a2 substrate (29). We assayed caffeine N3-demethylation by monitoring paraxanthine formation in liver membrane samples from WT and *Pgrmc1* KO mice (Fig. 3*C*). In the KO samples, paraxanthine formation was reduced by 30% at 50 μM caffeine. Taken together, these two enzyme assays demonstrate that loss of Pgrmc1 reduces Cyp1a2 activity, consistent with measured decreases in Cyp1a2 protein (Fig. 2, *B* and *C*).

We also tested the effect of loss of Pgrmc1 on Cyp2e1 activity. Cyp2e1 is a cytochrome P450 involved in the metabolism of ethanol, pharmaceutical drugs, and low-molecular-weight carcinogens, and p-nitrophenol is a known CYP2E1 substrate (1, 33). We assayed p-nitrophenol hydroxylation in liver membrane samples from WT and *Pgrmc1* KO mice (Fig. 3*D*). In the KO samples, p-nitrocatechol formation was reduced by 62% at 100 μM p-nitrophenol, consistent with reduced levels of Cyp2e1 protein in Pgrmc1 KO livers (Fig. 2, *B* and *C*). These activity assays show that loss of Pgrmc1 reduces cytochrome P450 activity, consistent with measured decreases in cytochrome P450 protein.

### Pgrmc1 protects against acetaminophen-induced liver injury

Since Pgrmc1 was required for maximal Cyp2e1 activity in liver membranes *in vitro,* we asked whether Pgrmc1 supports liver Cyp2e1 cytochrome P450 activity *in vivo*. Overdosing on the common over-the-counter analgesic acetaminophen (APAP) is the leading cause of acute liver failure in patients (34, 35). In 29% of APAP overdose cases, liver damage is so great that a liver transplant is required (35). Cyp2e1 metabolizes APAP, converting it to the reactive product N-acetyl-p-benzoquinone imine (NAPQI), which is subsequently glutathionylated for excretion (34). When normal doses of APAP are consumed, NAPQI is readily glutathionylated and no longer reactive (34). However, when overdoses of APAP are consumed, the Cyp2e1-dependent production of NAPQI overwhelms the glutathione pool and causes liver damage (34). *Cyp2e1* KO mice are protected against APAP-induced liver injury, which highlights the important role of Cyp2e1 in affecting APAP toxicity (36, 37). Flag-Pgrmc1 bound Cyp2e1 (Fig. 1*D*, Tables S2.1 and S2.2), and Cyp2e1 protein levels were 20% higher in WT liver than in *Pgrmc1* KO liver (Fig. 2, *B* and *C*).

**Figure 4:**
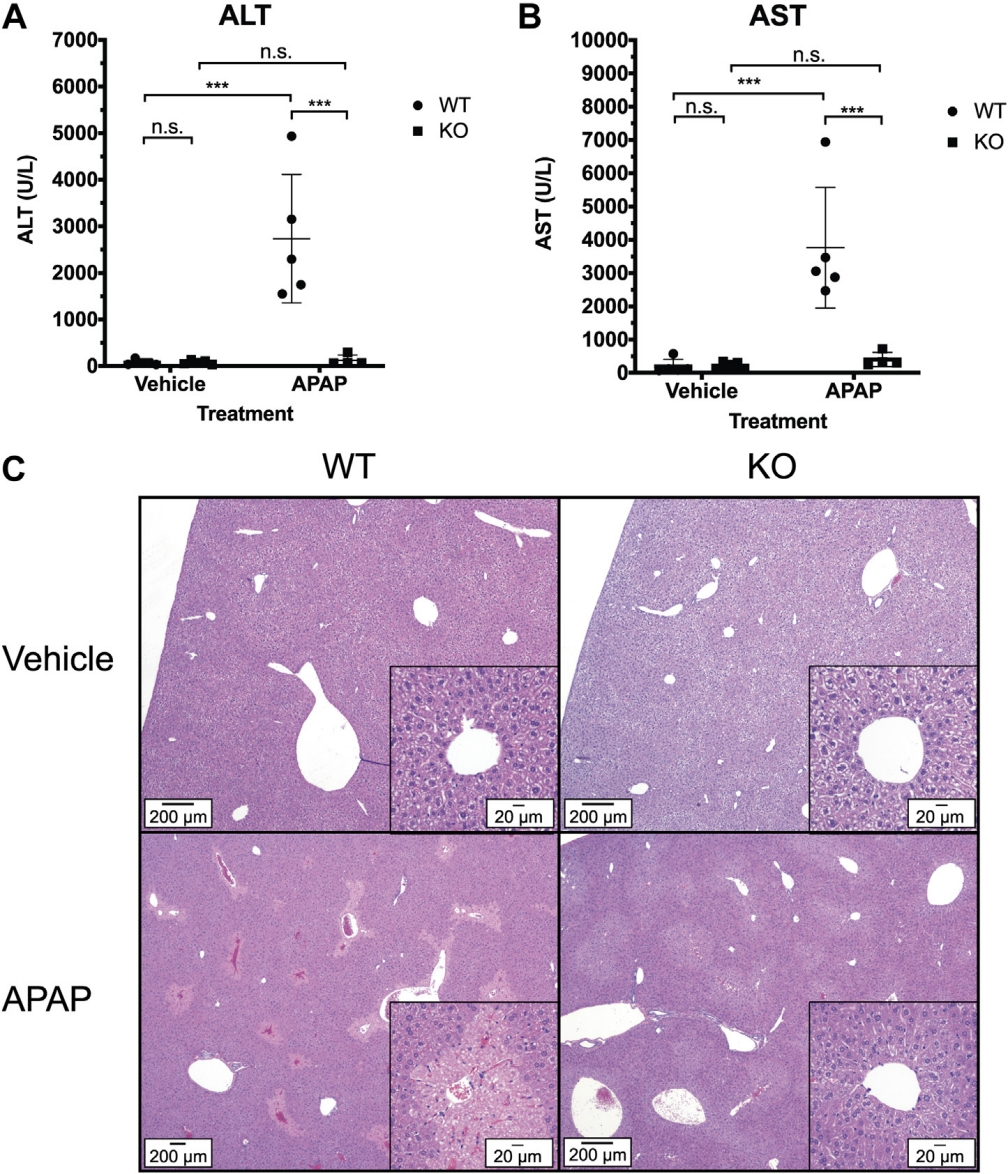
**Pgrmc1 protects against acetaminophen (APAP)-induced liver injury.***A* and *B*, serum markers of liver injury in *Pgrmc1* KO mice treated with APAP. After an overnight fast (16 h), mice were injected i.p. with 600 mg APAP/kg body weight in 50% DMSO/saline (v/v) and sacrificed 24 h after exposure. Serum ALT (A) and AST (*B*) were measured. Error bars are 1 SD (Vehicle: WT = 5 and KO = 5, APAP: WT = 5 and KO = 4; two-way ANOVA and Tukey’s HSD; “n.s.” is not significant, ∗∗∗ *p* ≤ 0.001). *C*, representative H&E stained sections of formalin fixed liver tissue from the mice in (*A*) and (*B*) showing a low magnification image of the liver section. Inset shows image around the central vein at high magnification. (Vehicle: WT = 5 and KO = 5, APAP: WT = 5 and KO = 5).

To investigate a functional role for Pgrmc1 in Cyp2e1 activity, we tested whether *Pgrmc1* KO protected against APAP-induced liver injury. Mice were injected with 600 mg APAP per kg body weight and euthanized 24 h later. All mice survived the study. Liver damage was surveyed by measuring serum ALT and AST and by histologic analysis of hematoxylin and eosin-stained liver sections. In WT animals treated with APAP, liver damage was apparent. ALT was elevated 37-fold, and AST was elevated 20-fold compared with vehicle-treated controls (Fig. 4, *A* and *B*). APAP-treated WT mice had centrilobular hepatocellular necrosis characteristic of APAP hepatotoxicity and cytoplasmic microvesicular vacuolation (Fig. 4*C*). Notably, *Pgrmc1* KO mice treated with APAP had the same ALT and AST levels as vehicle-treated mice (Fig. 4, *A* and *B*). While the APAP-treated KO mice exhibited cytoplasmic microvesicular vacuolation, they did not have the extensive centrilobular necrosis observed in APAP-treated WT mice (Fig. 4*C*). Thus, lack of Pgrmc1 expression was protective against the damage of APAP-induced liver injury, which is mediated by Cyp2e1. These data show that Pgrmc1-dependent stabilization of cytochromes P450 can have a clinically significant impact in a model of drug-induced liver injury.

### Y113F Pgrmc1 rescues cytochrome P450 levels in the liver

**Figure 5:**
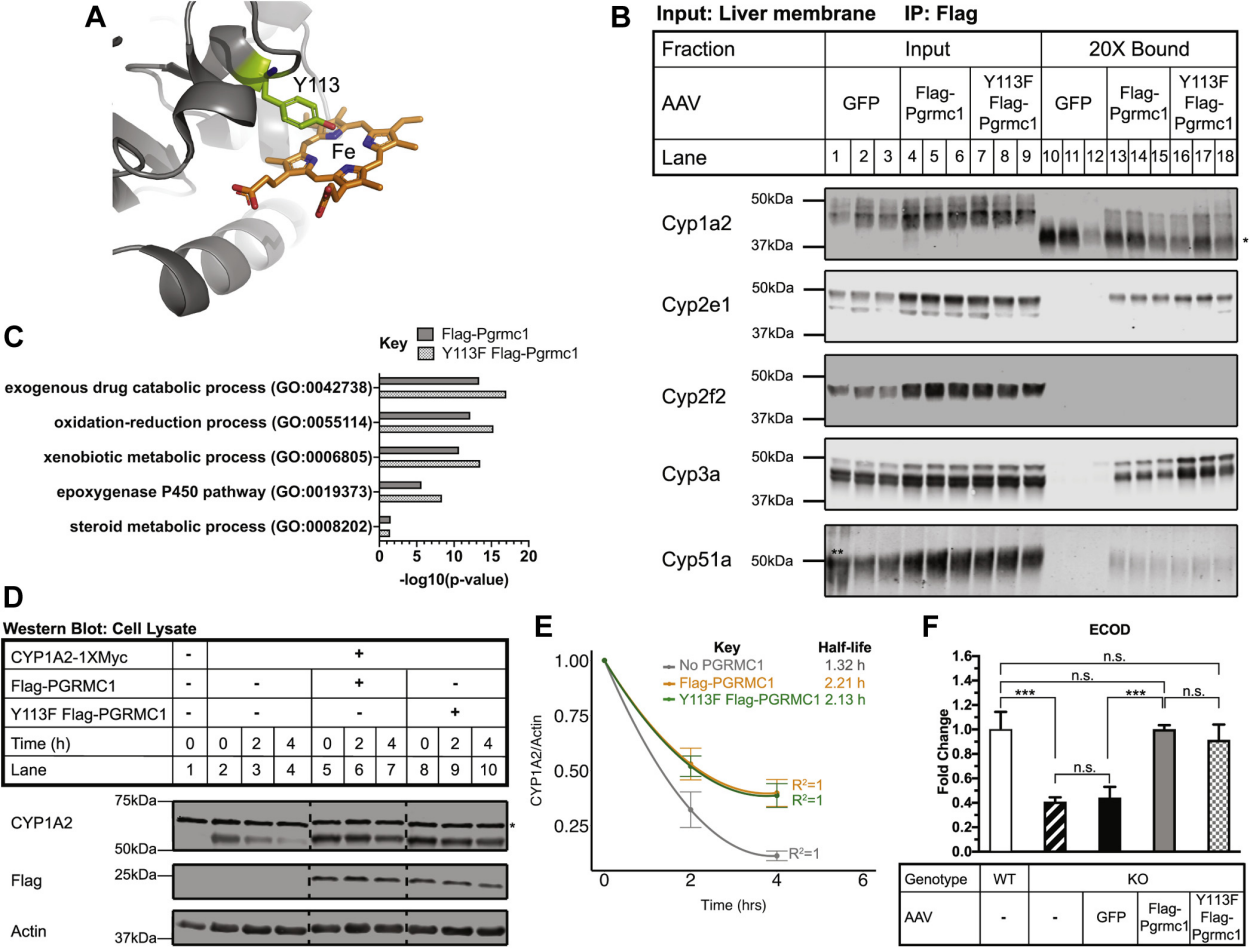
**Y113F PGRMC1 binds and stabilizes cytochromes P450.***A*, structure of truncated human PGRMC1 with heme axial ligand Y113 highlighted (PDB 4X8Y). *B*, input (1×) and bound (20×) fractions from Flag coimmunoprecipitation samples were subjected to western blotting for cytochromes P450 detected by mass spectrometry. Lanes 1 to 6 and 10 to 15 are identical to images in Figure 1*D* Lanes 1 to 6 and 7 to 12. (∗ denotes IgG; ∗∗ denotes ladder overflow into Lane 1). *C*, biological process GO Term analysis on candidate binding partners of Flag-Pgrmc1 and Y113F Flag-Pgrmc1 from liver. *Pgrmc1* KO mice were infected with AAV8 Y113F Flag-Pgrmc1 and processed as in Figure 1*C*. Y113F Flag-Pgrmc1 binding proteins were identified by mass spectrometry; 75 proteins have a fold change ≥20% compared with GFP. GO terms enriched in relation to the complete *M. musculus* proteome were identified using PANTHER. A Fisher’s exact test and Bonferroni correction were used to determine enriched GO terms with a *p*-value ≤ 0.05. *D*, *PGRMC1* KO cells were cotransfected with 5 μg CYP1A2-1XMyc and 10 μg of empty vector, Flag-PGRMC1, or Y113F Flag-PGRMC1 in a 10-cm plate. At 24 h posttransfection, cells were split 1:6 into a 6-well plate. At 48 h posttransfection, cells were treated with 100 μg/ml emetine and harvested every 2 h. Cell lysates were analyzed by western blotting. Actin is a loading control. Panels are representative of five independent experiments. Each panel is a montage from a single membrane with *dashed lines* denoting removed lanes. Lanes 1 to 7 are the same images as Figure 2*F* Lanes 1 to 7. (∗ denotes background band). *E*, the half-life of CYP1A2 in the presence and absence of Flag-PGRMC1 or Y113F Flag-PGRMC1 was determined from (*D*). Half-lives were calculated as in Figure 2*G*. Error bars are 1 SEM. (No PGRMC1 n = 5, Flag-PGRMC1 n = 5, Y113F Flag-PGRMC1 n = 5). *F*, ECOD reaction in liver membranes of *Pgrmc1* KO mice infected with AAV8 Y113F Flag-Pgrmc1. The reaction was conducted as in Figure 3*A* with 2 mM 7-ethoxycoumarin. Uninfected WT and KO samples were prepared as in Figure 3*A*. The infected KO samples were pooled samples from three male mice of each treatment group (AAV8 GFP, AAV8 Flag-Pgrmc1, AAV8 Y113F Flag-Pgrmc1) infected as in Figure 1*C*. Error bars are 1 SD (n = 3 technical replicates per condition; one-way ANOVA and Tukey HSD; “n.s.” is not significant, ∗∗∗ *p* ≤ 0.001).

The most notable biochemical property of PGRMC1 is its ability to bind heme. Residue Y113 was identified in the PGRMC1 crystal structure (PDB 4X8Y) as the axial iron-coordinating residue in the heme-binding pocket (Fig. 5*A*) (18). Kabe *et al.* reported that in *in vitro* binding assays with recombinant protein, CYP1A2 does not bind Y113F PGRMC1 lacking the N-terminal transmembrane domain. We tested whether this axial iron-coordinating residue was required for full-length Pgrmc1 to stabilize cytochromes P450. We infected *Pgrmc1* KO mice with AAV8 Y113F Flag-Pgrmc1, AAV8 GFP, or AAV8 Flag-Pgrmc1 as described above. We performed quantitative mass-spectrometry proteomics with isobaric tagging on membrane samples from these mice with three biological replicates per condition. A total of 624 proteins were detected in all three mice per condition, including 19 cytochromes P450 (Fig. S4, *A* and *B*, Tables S5.1–S5.6). Among the proteins with reduced abundance in the GFP sample compared with Flag-Pgrmc1 sample were Cyp1a2, Cyp2f2, Cyp51a, and Cyp2e1, which were also less abundant in *Pgrmc1* KO compared with WT mice (Fig. 2, *B* and *C*, Tables S5.1 and S5.2). These cytochromes P450 were also less abundant in the GFP samples compared with Y113F Flag-Pgrmc1 (Tables S5.3 and S5.4) . Similar to the trend observed for *Pgrmc1* KO mice (Fig. 2), cytochromes P450 tended to be less abundant in AAV8 GFP samples and more abundant in Flag-Pgrmc1 and Y113F Flag-Pgrmc1 samples (Fig. S4, *A* and *B*). Cytochromes P450 had no significant differential expression between Flag-Pgrmc1 and Y113F Flag-Pgrmc1 samples in the proteomics dataset (Fig. S4*C*, Tables S5.5 and S5.6). Protein expression of Cyp1a2, Cyp2e1, Cyp2f2, Cyp3a, and Cyp51a was confirmed to be lower in GFP samples compared with Flag-Pgrmc1 and Y113F Flag-Pgrmc1 samples by western blotting (Figs. 5*B* and S5*A*). Expression of Cyp1a2, Cyp2e1, Cyp3a, and Cyp51a was the same in the Y113F Flag-Pgrmc1 sample as the Flag-Pgrmc1 sample (Figs. 5*B* and S5*A*). Thus, the axial iron-coordinating residue is not required in mouse liver for Pgrmc1 to maintain cytochrome P450 levels.

We next tested whether Y113F Flag-Pgrmc1 binds cytochromes P450 in mouse liver by Flag coimmunoprecipitation coupled to mass spectrometry. Y113F Flag-Pgrmc1 bound 75 proteins (Table S2.2) , which was twice the number of binding partners for Flag-Pgrmc1. Among these 75 binding partners, there were 18 cytochromes P450. Binding of Y113F Flag-Pgrmc1 to Cyp1a2, Cyp2e1, Cyp3a, and Cyp51a was validated by western blotting (Figs. 5*B* and S5*B*). Consistent with the result for Flag-Pgrmc1, binding of Y113F Flag-Pgrmc1 to Cyp2f2 was not confirmed (Fig. 5*B*). The top enriched GO terms among the candidate binding partners were “exogenous drug catabolic process” (*p*= 9.96 × 10^−18^) and “oxidation-reduction process” (*p* = 5.45 × 10^−16^) and reflect the cytochromes P450 and additional proteins that are involved in electron transfer reactions (Fig. 5*C*). Flag-Pgrmc1 candidate binding partners were also enriched for these GO terms (Fig. 5*C*). Like Flag-Pgrmc1, Y113F Flag-Pgrmc1 binds cytochromes P450 and, more broadly, may bind enzymes involved in redox processes in the liver.

### Y113F PGRMC1 stabilizes CYP1A2 posttranslationally and supports Cyp1a2 activity in the liver

Since Y113F Flag-Pgrmc1 bound and affected steady-state protein levels of cytochromes P450 in the liver similar to Flag-Pgrmc1, we asked if Y113F Flag-PGRMC1 stabilized CYP1A2 in cell culture. We confirmed that CYP1A2-1XMyc bound Y113F Flag-PGRMC1 (Fig. S5*C*). Y113F Flag-PGRMC1 also stabilized CYP1A2-1XMyc to the same extent as Flag-PGRMC1 (Fig. 5, *D* and *E*). This experiment showed that the axial iron-coordinating residue of PGRMC1 is not required to stabilize CYP1A2.

Because Y113F Flag-PGRMC1 stabilized CYP1A2-1XMyc in human cells, and Y113F Flag-Pgrmc1 stabilized and bound Cyp1a2 in liver, we tested whether Y113F Pgrmc1 can also support Cyp1A2 activity in the liver. Using liver membrane fractions from *Pgrmc1* KO mice infected with AAV GFP, AAV Flag-Pgrmc1, or AAV Y113F Flag-Pgrmc1, we performed the ECOD metabolism assay at a 2 mM saturating substrate concentration (Fig. 5*F*). As expected, the AAV GFP sample generated the same amount of product as an uninfected *Pgrmc1* KO sample, and Flag-Pgrmc1 restored product formation to the level of the uninfected WT control (Fig. 5*F*). Notably, Y113F Flag-Pgrmc1 also restored product formation to the level of the uninfected WT control. Thus, the axial iron-coordinating residue Y113 of Pgrmc1 is not required for Pgrmc1 to support Cyp1a2 activity. Altogether, Y113F PGRMC1 behaved like wild-type PGRMC1 in all mouse liver and cell culture assays.

### Y113F Pgrmc1 is a heme-binding protein that does not bind ferrochelatase

**Figure 6:**
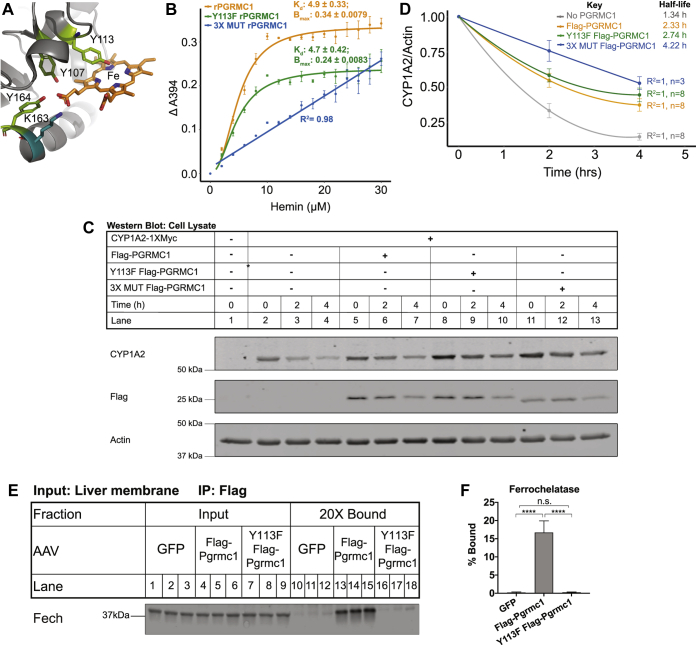
**Pgrmc1 binds cytochromes P450 in a heme-independent manner, while binding to ferrochelatase is sensitive to the Y113F mutation in PGRMC1.***A*, structure of truncated human PGRMC1 with heme ligands Y107, Y113, K163, and Y164 highlighted (PDB 4X8Y). *B*, heme-binding affinity of rPGRMC1, Y113F rPGRMC1, and 3X MUT rPGRMC1 protein. Each protein (10 μM) was incubated with 0 to 30 μM hemin for 16 h at room temperature. The amount of hemin bound was measured spectrophotometrically at A394. Data were fit to the Hill equation or a linear model as appropriate. *C*, 3X MUT Flag-PGRMC1 stabilizes CYP1A2 in human cells. *PGRMC1* KO cells were cotransfected with 5 μg CYP1A2-1XMyc and 10 μg of either empty vector, Flag-PGRMC1, Y113F Flag-PGRMC1, or 3X MUT Flag-PGRMC1 in a 10-cm plate. At 24 h posttransfection, cells were split 1:6 into a 6-well plate. At 48 h posttransfection, cells were treated with 100 μg/ml emetine and harvested every 2 h. Cell lysates were analyzed by western blotting. Actin is a loading control. Panels are representative of three independent experiments. *D*, the half-life of CYP1A2 in the presence and absence of Flag-PGRMC1, Y113F Flag-PGRMC1, or 3X MUT Flag-PGRMC1 was determined from (*C*) and Figure 5*D*. Half-lives were calculated as in Figure 2*G*. Error bars are 1 SEM. (No PGRMC1 n = 8, Flag-PGRMC1 n = 8, Y113F Flag-PGRMC1 n = 8, 3X MUT Flag-PGRMC1 n = 3). *E*, input (1×) and bound (20×) fractions from Flag coimmunoprecipitation samples were subjected to western blotting for ferrochelatase, which was detected by mass spectrometry to bind Flag-Pgrmc1 in *Pgrmc1* KO liver membranes. *F*, quantification of ferrochelatase in the bound fraction of the Flag coimmunoprecipitation for (*E*). Within each biological replicate, the ratio of cytochrome P450 expression in the Bound fraction to the Input fraction was quantified. Error is 1 SD (GFP n = 3, Flag-Pgrmc1 n = 3, Y113F Flag-Pgrmc1 n = 3); one-way ANOVA and Tukey HSD; n.s. denotes not significant, ∗∗∗∗*p* ≤ 0.0001).

Since Y113F Flag-Pgrmc1 binds and stabilizes cytochromes P450 like Flag-Pgrmc1, we investigated the ability of Y113F Pgrmc1 to bind heme *in vitro*. The crystal structure of human PGRMC1 revealed that four residues (K107, Y113, K163, Y164) coordinate heme in the binding pocket (Fig. 6*A*) (18). From *Escherichia coli,* we purified recombinant truncated 6X His-tagged human PGRMC1 (aa 43–195) as well as the mutants Y113F PGRMC1 and Y113F, K163A, Y164F (3X MUT) PGRMC1, and the affinity tag was removed by thrombin cleavage (Fig. S6*A*). After incubation with a 100-fold molar excess of hemin, recombinant PGRMC1 (rPGRMC1) had the deep reddish-brown color characteristic of a heme-binding protein (Fig. S6*B*). Y113F rPGRMC1 was a similar reddish-brown color (Fig. S6*B*), suggesting that it retained the ability to bind heme. Unlike rPGRMC1 and Y113F rPGRMC1, 3X MUT rPGRMC1 was much lighter in color, suggesting it has a lower binding affinity for heme than either rPGRMC1 or Y113F rPGRMC1 (Fig. S6*B*). To measure the heme-binding affinity of each rPGRMC1 protein, 10 μM of each protein was incubated with 0 to 30 μM hemin for 16 h. The amount of hemin bound was measured spectrophotometrically at A_394_. The data were fit to the Hill equation or a linear model as appropriate. rPGRMC1 bound heme with a K_d_ of 4.9 ± 0.33 μM (Fig. 6*B*), which is similar to the value reported for PGRMC2 (1.4 μM) (19). Y113F rPGRMC1 bound heme with the same affinity as rPGRMC1 (K_d_ = 4.7 ± 0.42 μM) (Fig. 6*B*). In contrast, 3X MUT rPGRMC1 bound heme nonspecifically (Fig. 6*B*). Taken together, these results indicate that mutation of the iron-coordinating residue Y113F in PGRMC1 does not prevent heme binding and that additional residues in the heme-binding pocket of PGRMC1 must be mutated to prevent heme binding.

Having identified a heme-binding mutant of PGRMC1, we next tested whether heme binding by PGRMC1 is required for PGRMC1 to bind and stabilize CYP1A2. Using *PGRMC1* KO cells, we found that 3X MUT Flag-PGRMC1 binds CYP1A2-1XMyc (Fig. S6*C*). Additionally, 3X MUT Flag-PGRMC1 stabilized CYP1A2-1XMyc in an emetine chase more effectively than Flag-PGRMC1 or Y113F Flag-PGRMC1 (Fig. 6, *C* and *D*). These results indicate that PGRMC1 binds and stabilizes CYP1A2 in a heme-independent fashion.

Despite the shared ability of PGRMC1 and Y113F PGRMC1 to bind and stabilize cytochromes P450 and their affinity for heme, we discovered one notable difference between Flag-Pgrmc1 and Y113F Flag-Pgrmc1. We performed a stringent quantitative analysis of the proteins that bound Flag-Pgrmc1 and Y113F Flag-Pgrmc1 in the Flag pull-down from liver membrane fractions (Tables S6.1–S6.9). As expected, Flag-Pgrmc1 and Y113F Flag-Pgrmc1 both bound cytochromes P450. Interestingly, ferrochelatase (Fech) bound Flag-Pgrmc1, but not Y113F Flag-Pgrmc1 (Fig. 6, *E* and *F*). Fech is responsible for the final step in heme synthesis (38), and binding between PGRMC1 and FECH has been reported previously in human cells (21). Binding had not previously been examined in mammalian liver. Binding of Fech to Flag-Pgrmc1 was robust, but Y113F Flag-Pgrmc1 failed to bind Fech despite equal expression (Fig. S6, *E* and *F*). Taken together, these results suggest that Y113F PGRMC1 is capable of binding heme with the affinity of the wild-type protein, but the axial iron-coordinating residue of Pgrmc1 is critical for Pgrmc1 binding to the heme biosynthetic enzyme ferrochelatase.

## Discussion

PGRMC1 is a membrane-bound, heme-binding protein implicated in a plethora of biological processes (16). Previous work demonstrated that PGRMC1 is a cytochrome P450-binding protein in mammalian cells, and PGRMC1 supports the enzymatic activity of CYP51A1 in cholesterol synthesis in this system (3). Here, we extend these findings to mammalian liver and show that PGRMC1 is broadly required for cytochrome P450 function *in vivo*. Using a whole-body *Pgrmc1* KO mouse, we observed (1) PGRMC1 binds many cytochromes P450 from diverse families in the liver; (2) PGRMC1 stabilizes cytochromes P450 posttranslationally in a heme-independent manner; (3) PGRMC1 is required for maximal activity in the liver of cytochrome P450 enzymes that it binds; (4) PGRMC1 alters cytochrome P450 activity at a physiologically significant level that may have a clinical impact; and (5) PGRMC1 binds the terminal heme synthesis enzyme Ferrochelatase (FECH) in the liver. Our observations expand knowledge on *in vivo* cytochrome P450 biology and implicate PGRMC1 in heme metabolism.

PGRMC1 binds many cytochromes P450 from diverse families in the liver. Pgrmc1 bound to 56% of the 23 cytochromes P450 assayed by mass spectrometry. We validated the binding interaction with Pgrmc1 for five cytochromes P450 (Cyp1a2, Cyp2e1, Cyp3a, Cyp51a1, Cyp2f2) by western blotting, and all interactions were confirmed, except Cyp2f2 for reasons that are unclear. PGRMC1 bound cytochromes P450 from families involved in different functions, including xenobiotic, pharmaceutical drug, cholesterol, and arachidonic acid metabolism (1). PGRMC1 may yet bind more cytochromes P450 than the 23 we report here. If the interaction between Pgrmc1 and cytochromes P450 is transient, which is likely since we measured that only 0.2 to 2% of the total amount of a cytochrome P450 bound Pgrmc1, then the full complement of cytochromes P450 that bind Pgrmc1 may not have been identified. Additionally, it is unknown whether PGRMC1 preferentially binds apo-cytochromes P450 or the heme-loaded forms, as such the protein synthesis rate and heme-loading rate of a cytochrome P450 may affect the duration of PGRMC1 binding and the ability to detect the interaction at steady state. Also, healthy laboratory mice come in contact with very few chemical stressors that would induce expression of cytochromes P450 that are not constitutively expressed and, therefore, the full complement of mouse P450 enzymes was not tested for Pgrmc1 binding. A structure of PGRMC1 bound to cytochromes P450 or chemical cross-linking studies would advance the field and potentially identify characteristics of the PGRMC1-cytochrome P450-binding interface that may allow for cytochrome P450-binding partners of PGRMC1 to be predicted.

PGRMC1 stabilizes cytochromes P450 posttranslationally. Cytochrome P450 proteins that bound PGRMC1 tended to be less abundant in *Pgrmc1* KO liver. Of the cytochromes P450 that bound Pgrmc1 for which we have complementary steady-state proteomic data, 64% (7/11) were less abundant in *Pgrmc1* KO liver. The decrease in protein expression cannot be attributed to a decrease in mRNA level as gene expression was either unchanged *(Cyp2j5*, *Cyp2c29*, *Cyp1a2*, *Cyp2e1*, *Cyp2f2*, *Cyp51a1)* or elevated (1.4-fold, *Cyp3a11*) in *Pgrmc1* KO liver. The elevation of cytochrome P450 transcripts in *Pgrmc1* KO liver, like *Cyp3a11*, may be a compensation mechanism to restore cytochrome P450 protein levels. Indeed, such a mechanism may actually mask the effect that loss of Pgrmc1 has on cytochrome P450 protein levels. For the three cytochromes P450 that were among the least abundant proteins in *Pgrmc1* KO mice (Cyp2f2, Cyp7b1, Cyp3a13), all three were shown to bind Pgrmc1 by at least one method (mass spectrometry or western blotting), and likewise their mRNAs were either unchanged (*Cyp2f2*, *Cyp3a13*) or elevated (twofold, *Cyp7b1*). Cyp1a2 bound Pgrmc1 and was less abundant in *Pgrmc1* KO liver. Using cultured cells, we confirmed that Flag-PGRMC1 bound CYP1A2 and stabilized CYP1A2 in a chase assay with a translation inhibitor. Collectively, these *in vivo* and *in vitro* data demonstrate that PGRMC1 binds and posttranslationally stabilizes cytochrome P450 enzymes in mouse liver.

PGRMC1 supports the activity of cytochrome P450 enzymes that it binds in mouse liver, including Cyp1a2 and Cyp2e1. The apparent maximal reaction rate (*V*_max_) in *Pgrmc1* KO microsomes for the Cyp1a2-dependent ECOD reaction was reduced by 40%, but there was no change in enzyme affinity (*K*_m_) (Fig. 3). Likewise, Cyp2e1 product formation was reduced by 62% in *Pgrmc1* KO mice, and the KO mice were protected from APAP-induced liver injury (Figs. 3 and 4) (36, 37). Since the *V*_max_ of an enzyme is proportional to the amount of enzyme present and the turnover of the enzyme (*k*_cat_), Pgrmc1 may affect either of these parameters to increase cytochrome P450 activity. Our data demonstrate that a decrease in Cyp1a2 and Cyp2e1 protein underlies the decrease in cytochrome P450 activity in *Pgrmc1* KO liver. Both Cyp1a2 and Cyp2e1 proteins bind Pgrmc1, and the proteins are expressed at lower levels in *Pgrmc1* KO liver. PGRMC1 most likely controls enzyme activity by stabilizing cytochromes P450 rather than affecting the catalytic cycle of the enzyme, but a concomitant effect on the *k*_cat_ of cytochromes P450 upon PGRMC1 binding cannot be ruled out as protein–protein interactions are also known to alter cytochrome P450 activity (39, 40, 41, 42). Evidence exists that PGRMC1 binds cytochromes P450 directly. PGRMC1/Dap1p bound the cytochromes P450 Erg11p and Erg5p directly in *S. pombe* (3)*,* and recombinant PGRMC1 bound CYP1A2 and CYP3A4 *in vitro* (18). No obvious adaptor protein was discovered among the candidate binding partners of PGRMC1 in this study. However, PGRMC2 cannot be ruled out as an adaptor, as binding of PGRMC1 and PGRMC2 has previously been reported and was observed by mass spectrometry in this study (19, 43). Given that Pgrmc1 supports the activity of Cyp1a2 and Cyp2e1, Pgrmc1 may increase the activity of other cytochromes P450 to which it binds and stabilizes. It is unknown if binding of PGRMC1 to cytochromes P450 is regulated. If true, regulated binding would represent a previously unappreciated mechanism for regulation of cytochrome P450 protein levels and activity.

While the mechanism by which PGRMC1 maintains cytochrome P450 protein levels is unknown, this interaction alters cytochrome P450 activity in a physiologically significant way that can have a clinical impact. In *Pgrmc1* KO mice, systemic cholesterol is 8% lower than controls, which is likely due to a Pgrmc1-dependent effect on Cyp51a1 in the cholesterol biosynthetic pathway. Hughes *et al* (3)*.* demonstrated that PGRMC1 binds CYP51A1 and supports cholesterol synthesis in HEK293 cells. Recently, deletion of *PGRMC1* was shown to cause X-linked isolated pediatric cataract in humans, and the authors proposed that this is due to disruptions in CYP51A1-dependent cholesterol synthesis in the lens (44). The fact that *Pgrmc1* KO mice are protected from APAP-induced toxicity demonstrates that Pgrmc1 has an *in vivo* effect on cytochrome P450 activity. PGRMC1 also affects the stability of CYP3A and other cytochromes P450 involved in the metabolism of pharmaceutical drugs, which suggests that PGRMC1 may play a clinically significant role in drug metabolism in patients. Individuals with mutations or polymorphisms of PGRMC1 should be studied for a role in drug metabolism or other disease phenotypes associated with cytochrome P450-dependent synthesis reactions. Consistent with the presence of a subclinical phenotype in *Pgrmc1* KO mice, KO livers had increased levels of core proteasome subunits associated with membranes (Table S3.3) and increased mRNA expression for the acute-phase response proteins, SAA1 and SAA2 (Table S4.3). The full scope of PGRMC1 function in P450 biology is unknown since pathways may need to be stressed in order to observe a phenotype as seen for Cyp2e1and APAP overdose.

The axial heme coordinating residue Y113 of PGRMC1 is not required for PGRMC1 to bind heme or to bind and stabilize cytochromes P450 (Figs. 5 and 6). This axial residue was not definitively identified until 2016 when Kabe *et al*. (18) solved the X-ray crystal structure of truncated, recombinant PGRMC1 and identified Y113 as the elusive residue. While this previous study showed that Y113 is required for PGRMC1 to form a homodimer *in vitro* by mutating the tyrosine to the structurally similar phenylalanine residue, the authors did not report the heme-binding affinity of the Y113F mutant. Our work shows that Y113F PGRMC1 binds heme specifically and with similar affinity as PGRMC1. Kabe *et al.* (18) reported the Y113F PGRMC1 mutant does not bind CYP1A2 in an *in vitro* binding experiment that used a truncated form of PGRMC1 (aa 44–195). Contrary to this result, our work shows that full-length Y113F PGRMC1 binds CYP1A2 in both cultured cells and mouse liver and that Y113F PGRMC1 promotes CYP1A2 stability as well as wild-type PGRMC1. In fact, Y113F Pgrmc1 binds all 13 cytochromes P450 that Pgrmc1 binds in mouse liver plus an additional five cytochromes P450. These data suggest that the N-terminal transmembrane domain of PGRMC1 may contribute to cytochrome P450 binding.

To render PGRMC1 unable to bind heme, Y113 and two additional residues that coordinate the protoporphyrin ring (K163, Y164) must be mutated. This 3X MUT PGRMC1 bound CYP1A2 and stabilized it in cultured cells (Figs. 6 and S6), demonstrating that PGRMC1 can bind and stabilize cytochromes P450 in a heme-independent fashion. Mechanisms of cytochrome P450 turnover are relatively understudied compared with cytochrome P450 enzymology, even though turnover influences cytochrome P450 activity (45, 46). One intriguing hypothesis is that binding of PGRMC1 blocks ubiquitination sites on cytochromes P450 delaying their turnover. If this is the case, the binding between cytochromes P450 and PGRMC1 would not require PGRMC1 to bind heme. Interestingly, Pgrmc1 and Y113F Pgrmc1 also bound other redox proteins in the liver that are not heme-binding proteins (Tables S2.1 and S2.2). This opens the possibility that Pgrmc1 may stabilize these redox proteins as well. The mechanism of the heme-independent stabilization of cytochromes P450 by PGRMC1 and any heme-dependent function of PGRMC1 remain to be elucidated.

PGRMC1 binding to heme is not required for cytochrome P450 binding or stability. However, the Y113F mutant that removes the iron-coordinating hydroxyl group disrupts binding to ferrochelatase (FECH) in mouse liver. As FECH is the terminal enzyme in mitochondrial heme synthesis (38), this observation opens up questions about the role of PGRMC1 in heme metabolism. Piel *et al.* previously reported that PGRMC1 binds to FECH in human embryonic kidney and leukemia cells (21). Here, we extended this observation to mouse liver. Roughly 20% of the liver Fech present bound to Pgrmc1, suggesting a potentially direct interaction. The precise mechanism by which heme is transferred safely from FECH to hemoproteins throughout the cell remains to be elucidated (38). In the cellular environment, heme is a highly reactive molecule that must be sequestered to prevent indiscriminate cellular damage and must be delivered safely to heme-binding proteins (38). The Y113 residue is critical for PGRMC1 to bind FECH. Although we cannot rule out that small differences in heme binding affinity underlie the dramatic change in Pgrmc1-Fech binding (Fig. 6), a more likely interpretation is that the Y113 residue is structurally important for the interaction of the two proteins. Others have hypothesized that FECH transfers heme to PGRMC1 (19, 21). To this hypothesis, we add that heme transfer is likely Y113-dependent. In this regard, detailed structural information of the PGRMC1-FECH complex would advance the field. Interestingly, the NCBI dbSNP database contains 1751 SNPs for *PGRMC1* of which 96 (5.5%) are missense mutations. No missense mutations occur in the Y113 codon or those of the other three residues that compose the heme-binding site (Fig. 6), consistent with these PGRMC1 residues having an important function *in vivo*.

The binding of PGRMC1 to FECH, which is associated with the inner membrane in the mitochondrial matrix, and ER-localized cytochrome P450 enzymes raises the question of how PGRMC1 localizes to these different subcellular compartments and whether its localization is dynamic. The binding of PGRMC1 and FECH places PGRMC1 in a key position to transfer heme throughout the cell. It is difficult to explain how an ER-resident Type 1 membrane protein like PGRMC1 can interact with FECH, which resides in the mitochondrial matrix associated with the inner membrane, such that heme from FECH could be transferred to the cytochrome-b5 domain of PGRMC1. While studies have described PGRMC1 as localized to many different subcellular compartments, no model of the PGRMC1-FECH interaction has yet provided a satisfactory explanation that respects the rules of protein trafficking and membrane topology (16, 19, 21, 47). Additional studies on the subcellular localization of PGRMC1 are necessary; two populations of PGRMC1 may exist (ER and mitochondria) or PGRMC1 may reside in the ER at ER–mitochondria contact sites. PGRMC1 may deliver FECH-derived heme to apo-hemoproteins directly and/or to a heme chaperone, one of which may be PGRMC2. PGRMC2 or a yet unidentified protein may be the intermediary heme chaperone receiving the FECH-derived heme from PGRMC1 and conveying it to apo-hemoproteins, including cytochromes P450, in the cytosol and ER.

Galmozzi *et al.* recently showed that the PGRMC1 paralog, PGRMC2, is a heme chaperone, which suggests that PGRMC1 may also be a heme chaperone (19). In support of this hypothesis, Piel *et al.* demonstrated that recombinant PGRMC1 can donate heme to apo-cytochrome b5 (21). Further, cells depleted of PGRMC1 by shRNA had reduced levels of labile heme in mitochondria, ER, nucleus, and cytosol (19). Depletion of PGRMC2 by shRNA reduced labile heme only in the mitochondria and nucleus (19). Notably, PGRMC1 was epistatic to PGRMC2 in this assay, indicating that PGRMC1 acts upstream of PGRMC2. Based on these observations and PGRMC1 binding to FECH (21), Galmozzi *et al.* propose that PGRMC1 accepts heme from FECH, passing it off to PGRMC2 for delivery to the nucleus and the nuclear hormone receptor Rev-Erbα (19). Our data support a role for PGRMC1 in accepting heme from FECH given that loss of heme iron coordination completely disrupts FECH binding. However, our mouse KO studies indicate that if Pgrmc1 is a heme chaperone *in vivo*, it cannot be the only mechanism for newly synthesized heme transport as *Pgrmc1* KO mice are alive. In addition to the model above, it is possible that PGRMC1 chaperones heme to cytochromes P450. Yet based on our data, the inability of PGRMC1 to transfer heme would not impact cytochrome P450 stability. Our findings that PGRMC1 stabilizes cytochrome P450 enzymes in a heme-independent manner demonstrate that PGRMC1 has at least two functions in the cell.

Altogether, this study highlights that PGRMC1 has a significant impact on cytochrome P450 activity and physiology *in vivo* and has a second function in heme metabolism. As noted in the introduction, PGRMC1 has been implicated in a wide range of biological activities. Given the large number of cytochromes P450 bound by PGRMC1, investigators should examine whether defects in cytochrome P450 activity underlie these observations. Here, we examined the role of PGRMC1 in cytochromes P450 activity in mouse liver and present the first mouse tissue interactome for Pgrmc1-binding partners. Pgrmc1 represents 2% of mouse liver protein (48), and we identified many noncytochrome P450-binding partners, such as redox proteins, atlastin, and BAP31 (49). These proteins do not bind heme, suggesting that additional PGRMC1 functions remain to be discovered. Finally, PGRMC1 was recently identified as the causative mutation in X-linked isolated pediatric cataract (44). In addition to confirming that defects in CYP51A1 underlie this disease, careful examination of individuals with PGRMC1 mutations may reveal additional phenotypes associated with other cytochromes P450.

## Experimental procedures

### Materials

Unless otherwise stated, we obtained common reagents from Thermo Fisher. Unless otherwise stated, chemicals were obtained from Sigma.

### Plasmids

The mammalian expression vector, Flag-PGRMC1, encoding human *PGRMC1* (NM_006667.5) with a single N-terminal Flag tag in the vector pcDNA3.1 was previously described (3). The mammalian expression vector Y113F Flag-PGRMC1 was generated from the Flag-PGRMC1 mammalian expression vector by site-directed mutagenesis (NM_006667.5 nucleotide 415 A->T). The mammalian expression vector Y113F, K163A, Y164F (3X MUT) Flag-PGRMC1 was generated from the Y113F Flag-PGRMC1 mammalian expression vector by site-directed mutagenesis (NM_006667.5 nucleotides 1585–1590 AAGTAT->GCGTTT).

The mammalian expression vector, CYP1A2-1XMyc, encoding human *CYP1A2* was generated by subcloning bases 63 to 1608 of the *CYP1A2* CDS (NM_000761.5) from Invitrogen Ultimate ORF IOH52560 (Johns Hopkins University High Throughput Biology HiT Center) to pcDNA3.1. The sequence contains a point mutation (C1200T) that is nonsynonymous (NP_000752.2, S380P). CYP1A2 is tagged at the C-terminus with a single Myc tag. The GP78-5X Myc plasmid was a gift of Dr Russell DeBose-Boyd (50).

The plasmids for the production of AAV8 viral particles encoding murine Pgrmc1 were generated by replacing the *EGFP* cassette in an AAV8 EGFP plasmid (AV-8-0101, University of Pennsylvania Vector Core) with *Flag-Pgrmc1* or *Y113F Flag-Pgrmc1* subcloned from the mammalian expression vectors described here: The mammalian expression vector, Flag-Pgrmc1, encoding murine *Pgrmc1* was generated by subcloning the *Pgrmc1* CDS (NM_016783.4 nucleotides 95–682) to pcDNA3.1. Pgrmc1 was tagged at the N-terminus with a single Flag tag. The mammalian expression vector Y113F Flag-Pgrmc1 was generated from the Flag-Pgrmc1 mammalian expression vector by site-directed mutagenesis (NM_016783.4 nucleotide 432 A->T). AAV8 EGFP, AAV8 Flag-Pgrmc1, and AAV8 Y113F Flag-Pgrmc1 viral particles were produced by the University of Pennsylvania Vector Core.

The bacterial expression vectors for truncated human PGRMC1 and Y113F PGRMC1 were generated by subcloning the sequence for amino acids 43 to 195 (NP_006658.1) from the Flag-PGRMC1 and Y113F Flag-PGRMC1 mammalian expression vectors into pET28a (EMD BioScience). The bacterial expression vector for human Y113F, K163A, K164A (3X MUT) PGRMC1 was generated by PCR mutagenesis (NM_006667.5 nucleotides 1585–1590 AAGTAT->GCGTTT) of the Y113F PGRMC1 bacterial expression vector.

### Cell line generation

A *PGRMC1* KO cell line from the human fibroblast cell line SV589 (gift of Drs Michael Brown and Joseph Goldstein) was generated by clustered regularly interspaced short palindromic repeats (CRISPR)-Cas9-mediated genome editing (30). SV589 and *PGRMC1* KO cells were grown in DMEM with L-glutamine, 4.5 g/l glucose, and sodium pyruvate (Corning 10013CV); 10% (v/v) FBS; and penicillin-streptomycin (100 U/ml, Gibco) at 37 °C with 5% CO_2_. Human *PGRMC1* (NM_006667.5) contains three exons and is translated into a 195-aa protein. A CRISPR guide RNA (gRNA) to target sequence nucleotides 114 to 133 (5′-GCTCTCCAGATCGCTTGGGT-3′) located in exon 1 was cloned into the Cas9-gRNA vector PX459 (Addgene #48139) (51). To generate the *PGRMC1* knockout line, the Cas9-gRNA plasmid targeting *PGRMC1* was transfected in SV589 cells using Polyfect transfection reagent (Qiagen). Transfected SV589 cells were selected for growth in the presence of 1 μg/ml puromycin (P8833, Sigma) for 3 days. Single clones were isolated by dilution cloning. Genomic DNA flanking the gRNA target site was amplified by standard PCR, then sequenced by Sanger sequencing. Primer sequences were human *PGRMC1* forward: 5′-CTCCCAGGTAGAACTGAG-3′ and reverse 5′-CACATC GAACACCTTGCC-3′. One isolated clone contained a 10 bp deletion and an 11 bp deletion in both *PGRMC1* alleles. Knockout of *PGRMC1* was further confirmed by immunoblotting.

### Tissue culture

For validation of the *PGRMC1* KO cell line, 1 × 10^6^ SV589 or 2 × 10^6^ *PGRMC1* KO cells were plated in 10-cm plates. The next day, the cells were transfected with 15 μg plasmid DNA (15 μg pcDNA 3.1 or 10 μg Flag-PGRMC1 plus 5 μg pcDNA3.1), with 50 μl Polyfect (Qiagen) according to manufacturer’s instructions for HeLa cells. The cells were harvested 24 h after transfection. For chase experiments, 1.6 × 10^6^ *PGRMC1* KO cells were seeded in a 10-cm plate. Two days after seeding, cells were transfected with a total of 15 μg plasmid DNA (10 μg Flag-PGRMC1 or Y113F Flag-PGRMC1 or 3X MUT Flag-PGRMC1, 5 μg CYP1A2-1XMyc, and/or pcDNA3.1), with 50 μl Polyfect (Qiagen) according to manufacturer’s instructions for HeLa cells. After 24 h, the cells were split at 1:6 to a 6-well plate. At 48 h posttransfection, the cells were treated with 100 μg/ml emetine (Sigma) for 4 h. For Flag coimmunoprecipitation, 1.5 × 10^6^ *PGRMC1* KO cells were seeded in a 10-cm plate. The next day the cells were transfected with 15.05 μg plasmid DNA (10 μg Flag-PGRMC1 or Y113F Flag-PGRMC1 or 3X MUT Flag-PGRMC1, 5 μg CYP1A2-1XMyc, 0.05 μg GP78-5XMyc, and/or pcDNA3.1) with 50 μl Polyfect (Qiagen) according to manufacturer’s instructions for HeLa cells. Cells were harvested 24 h after transfection and used immediately.

### Animal husbandry

The Johns Hopkins animal care and use program is accredited by AAALAC international, and the Johns Hopkins Institutional Animal Care and Use Committee (IACUC) reviewed and approved all procedures. Routine health surveillance using dirty bedding sentinel serology indicated that the mice were free of the following organisms: mouse hepatitis virus, minute virus of mice, mouse parvovirus, epizootic diarrhea of infant mice (rotavirus), Theilers murine encephalomyelitis virus, murine norovirus, Sendai virus, pneumonia virus of mice, reovirus, lymphocytic choriomeningitis virus, ectromelia virus, mouse adenovirus (FL & K87), mouse cytomegalovirus, *Mycoplasma pulmonis*, fur mites, and pinworms. Mice were housed in social groups (2–5 mice) of the same sex in individually ventilated cages (Allentown Caging Inc) with autoclaved corncob bedding (Teklad, Envigo) and nesting material (Animal Specialties and Provisions). Cages were changed every 14 days. Autoclaved feed (Teklad Global 2018S) was provided *ad libitum*, and water was provided *via* in-cage automated watering systems (Systems Engineering, Inc). The room was maintained at 22 ± 1 °C on a 14:10 light:dark cycle at 40 to 70% humidity. Euthanasia was performed under isoflurane anesthesia by cervical dislocation. Whole blood was collected *via* cardiocentesis and placed in a heparin-coated green-top tube for plasma separation or a yellow-top tube with serum separator gel for serum separation (BD Biosciences). Clinical chemistry was performed with a Vet AceTM analyzer (Alfa Wassermann). Complete Blood Count (CBC) was performed with a Procyte Dx (Idexx Laboratories Inc). After harvest, liver tissue was flash frozen in liquid nitrogen for molecular analysis or stored in 10% neutral buffered formalin (Sigma) for histology.

### Generating PGRMC1 knockout mice

*Pgrmc1* floxed mice (C57BL/6N) were generated by inGenious Targeting Laboratory (iTL). The long homology arm of the targeting construct was 889 to 6921 bp upstream of exon 1 (Ensembl GRCm38 X chromosome), and the short homology arm was 296 to 2102 bp downstream of exon 2. The targeting construct inserted loxP sites 568 bp upstream of exon 1 and 1906 bp downstream of exon 2. The targeting construct also contained a neomycin cassette flanked by FRT sites within the loxP sites. Targeted iTL IC1 (C57BL/6N) male embryonic stem cells were microinjected into Balb/c blastocysts. Resulting chimeras with a high percentage black coat color were mated to C57BL/6N mice to generate F1 heterozygous offspring. F1 female heterozygotes were crossed to male Sox2-Cre mice (B6.Cg-Tg(Sox2-cre)1Amc/J, Jackson Laboratories) at Johns Hopkins animal facilities. The resulting whole-body *Pgrmc1* knockout (KO) mice lacking exons 1 and 2 were bred for this study. Male mice aged 8 to 12 weeks were used for this study, except for the acetaminophen-induced liver injury study where male mice aged 13 to 15 weeks were used.

### Generating anti-human PGRMC1 antibody

Hexahistidine-tagged recombinant human PGRMC1 (amino acids 43–195) antigen was purified from *E. coli* using Ni2+-NTA agarose (Qiagen). PGRMC1 antiserum (5944) was generated by Covance using a standard protocol.

### Liver and cell lysate preparation

To make liver lysates, liver tissue was disrupted with a Tissue Lyser (Qiagen) and steel beads in the presence of RIPA buffer (50 mM Tris-HCl, pH 8.0, 150 mM NaCl, 1% Igepal (v/v), 0.5% (w/v) sodium deoxycholate, 0.1% (w/v) SDS) plus 1X protease inhibitors (Protease Complete, Roche) at 4 °C. Cell lysates were made by resuspending cell pellets in RIPA buffer (approximately five pellet volumes) with protease inhibitors (0.5 μM phenylmethylsulfonyl fluoride, 5 μg/ml Pepstatin A, 10 μg/ml Leupeptin) on ice. Lysates were cleared by spinning in a microfuge at 21,130*g* for 10 to 15 min at 4 °C, and the supernatant was processed as the sample.

### Liver membrane preparation

Liver membrane fractions were prepared as described previously (52) with some minor modifications. Frozen tissue (∼1 g) was thawed on ice in five tissue volumes of Homogenization Buffer (100 mM Tris-HCl, pH 7.4, 100 mM KCl, 100 mM EDTA) and homogenized with an immersion blender (Bamix) until homogenization was completed based on visual inspection of the sample. The homogenate was cleared by spinning at 10,000*g* for 30 min at 4 °C. The supernatant was spun at 100,000*g* for 90 min at 4 °C. The pellet was washed with Resuspension Buffer (100 mM sodium pyrophosphate, pH 7.4, 1 mM EDTA) and spun at 100,000*g* for 60 min at 4 °C. The pellet was defined as the enriched membrane fraction. It was resuspended in Storage Buffer (50 mM potassium phosphate, pH 7.4, 0.1 mM EDTA, 20% (v/v) glycerol) with 0.1 mM DTT to 10 to 20 mg protein/ml. DTT was omitted for samples intended for coimmunoprecipitation. All buffers contained protease inhibitors (Protease Complete, Roche) at 1X or at 2X for samples intended for coimmunoprecipitation.

### Immunoblotting

Protein concentration in lysates and membrane fractions was measured using the BCA kit (Pierce), and samples were mixed with 5X SDS loading buffer (150 mM Tris-HCl, pH 6.8, 15% (w/v) SDS, 25% (v/v) glycerol, 0.2% (w/v) bromophenol blue) with or without 12.5% (v/v) β-mercaptoethanol (BME). After heating at 65 °C for 10 to 15 min, proteins were subjected to SDS-PAGE and transferred to nitrocellulose membranes (BioRad). The membranes were blocked with 5% (w/v) dried milk in PBS-T [PBS +0.05% (v/v) Tween 20], then incubated with primary antibodies. Primary antibodies and working concentrations included: rabbit anti-PGRMC1 5944 (1:500–1:1000), mouse anti-Cyp1a2 (D15) (sc53241, Santa Cruz, 1:100), rabbit anti-Cyp2e1 (ab28146, Abcam, 1:1000), rabbit anti-CYP3A4 (PA1-343, Thermo Fisher, 1:1000), mouse anti-β-Actin (C4) (sc-47778, Santa Cruz, 1:1000), mouse anti-c-Myc (9E10) (sc-40, Santa Cruz, 1:1000), rabbit anti-GFP (NB600–308, Novus, 1:1000–1:4000), rabbit anti-calnexin (20888, EMD Millipore, 1:1000–1:2000), rabbit anti-Cyp2F2 (gift of Dr Xinxin Ding, 1:1000) (53), rabbit anti-Cyp7b1 (gift of Dr David Russell, 1:1000) (54), rabbit anti-Cyp51 (gift of Dr Damjana Rozman, 1:1000) (55), and mouse anti-Ferrochelatase (A-3) (sc377377, Santa Cruz, 1:500). Incubations with primary antibody were performed overnight at 4 °C unless otherwise noted. Bound antibodies were visualized with IRDye800CW or IRDye680RD mouse or rabbit IgG detection reagent (LI-COR, 1:20,000) with one exception. For western blots of samples from Flag coimmunoprecipitation probed with the anti-Cyp1A2 antibody, a Quick Western Kit was used according to manufacturer’s instructions (LI-COR). Quantification of western blot signals was performed using Image Studio software (LI-COR). Images exported from Image Studio were adjusted for image rotation and brightness and contrast across the whole image using Adobe Photoshop.

### Flag coimmunoprecipitation

Flag pull-downs from mouse liver were performed on samples from male mice sacrificed 8 days after tail vein infection with 5 × 10^11^ particles of AAV8 GFP, Flag-Pgrmc1, or Y113F Flag-Pgrmc1. All buffers contained protease inhibitors (2X Protease Complete, Roche). One milligram of liver membrane protein in 150 μl microsome Storage Buffer (6.67 mg/ml) was diluted to a final concentration of 2 mg/ml in 1 mM MgCl_2_. The sample was treated with ten units of benzonase (EMD Millipore) on ice for 30 min. Next, an equal volume of 2X TAP lysis buffer [12 mM Na_2_HPO_4_, 8 mM NaH_2_PO_4_, pH 7.5, 150 mM NaCl, 4 mM EDTA, 2% (w/v) n-Dodecyl-β-D-maltoside (DDM, ACROS Organics)] was added to dilute the sample to 1 mg/ml. Insoluble material was removed by centrifugation at 20,000*g* for 10 to 15 min at 4 °C. Flag-M2 agarose (Sigma) was washed once in three bead volumes of 1X TAP lysis buffer containing 0.715 mM MgCl_2_. Then, beads were blocked in 20 bead volumes of wild-type liver lysate at 1 mg/ml prepared from a 12-week-old, wild-type male mouse in 1X TAP lysis buffer. The beads were blocked for 1 h at 4 °C and then washed three times in ten bead volumes of 1X TAP lysis buffer containing 0.715 mM MgCl_2_. Each sample was incubated with 25 μl Flag M2 beads for 1 h at 4 °C while rotating. The beads were washed 3X in 20 bead volumes of 1X TAP lysis buffer. The fourth and final wash was done in 20 bead volumes of 1X TAP lysis buffer containing 0.1% (w/v) DDM, and the samples transferred to a fresh tube. The bound fraction was eluted by incubating the beads at 65 °C for 10 min in 50 μl of elution buffer (30 mM Tris-HCl, pH 7.5, 0.125% (w/v) SDS). For mass spectrometry analysis of Pgrmc1-binding proteins, the Flag coimmunoprecipitation was performed in technical triplicate for each biological replicate (three each for GFP, Flag-Pgrmc1, Y113F Pgrmc1). Equal volumes of eluate from each technical replicate were combined to form a pooled sample for each biological replicate that was subsequently analyzed by mass spectrometry. For mass spectrometry of the membrane proteome of AAV8 GFP, Flag-Pgrmc1, or Y113F Flag-Pgrmc1 infected *Pgrmc1* KO liver, the samples were prepared as described above with benzonase treatment and diluted to 1 mg/ml before sonication.

For Flag pull-downs from transfected cultured cells, cells were lysed in 1X TAP lysis buffer with protease inhibitors (0.5 μM phenylmethylsulfonyl fluoride, 5 μg/ml Pepstatin A, 10 μg/ml Leupeptin). Lysates were solubilized by rotating at 4 °C for 1 h and cleared by spinning in a microfuge at 20,000*g* for 10 to 15 min at 4 °C. Flag-M2 agarose (Sigma) was washed once in six bead volumes of 1X TAP lysis buffer. Then, the beads were blocked in six bead volumes of 3% BSA (w/v, Sigma) in 1X TAP lysis buffer. The beads were blocked for 45 to 60 min at room temperature and then washed twice in six bead volumes of 1X TAP lysis buffer. Each sample (300 μg protein) was incubated with 10 μl Flag M2 agarose (Sigma) at a protein concentration of 1 mg/ml for 1 h at 4 °C while rotating. The beads were washed 3X in 30 to 50 bead volumes of 1X TAP lysis buffer. After the second wash, the samples were transferred to a fresh tube. The bound fraction was eluted by incubating the beads at 65 °C for 10 min in 30 μl of 1X SDS loading buffer without BME.

### Histology

Tissues fixed in 10% neutral buffered formalin (Sigma) were processed, paraffin embedded, sectioned (4 μm), and stained with hematoxylin and eosin according to standard protocols by Oncology Tissue Services (Johns Hopkins University). Slides were viewed on a Nikon Eclipse Ci microscope and images captured using Nikon DS-Fi2 camera. Images were edited for white balance across the whole image using Adobe Photoshop.

### Metabolite analysis with ^1^H-nuclear magnetic resonance spectroscopy

Mice were fasted for 4 h before sacrifice around 1:00 PM (ZT-7). Liver tissue (approximately 300 mg) was snap frozen in liquid nitrogen and processed the same day. Briefly, the tissue was homogenized in two tissue volumes of 20 mM phosphate buffer, pH 7.4. To the supernatant, four tissue volumes of methanol were added, and the samples vortexed before incubation at −20 °C for 30 min. The samples were spun at 13,000*g* at 4 °C for 15 min. The supernatant was dried in a speed vac overnight, and pellets were saved for protein quantification. Dried samples were constituted with 20 mM phosphate buffer containing 0.1 mM trimethylsilylpropionic acid (TMSP) and 0.1 mM NaN_3_. Proton NMR spectra were acquired, analyzed, and quantified as previously described (56).

### Mass spectrometry and proteomic analysis

To quantify protein levels in *Pgrmc1* KO livers, quantitative proteomics was employed. Equal masses of liver membrane protein from four WT and four KO mice (Replicate #1) or five WT and five KO mice (Replicate #2) were pooled by genotype to produce two biological replicates per genotype. In each MS run, a WT and a KO pool were labeled with three different isobaric tags, resulting in three technical replicates per genotype per experiment. The samples were digested with the protease trypsin, which specifically cleaves at the carboxyl side of the amino acids lysine and arginine. Next, the samples were labeled with unique Tandem Mass Tag (TMT) 10-plex reagent (Thermo Fisher) according to the manufacturer’s protocol. The combined labeled sample was cleaned up from excess TMT tag with a detergent removal spin column (Pierce).

The labeled sample was fractionated with five or four basic, reversed-phase fractions of 5%, 15%, 20%, 30%, and 75% or 5%, 15%, 25%, and 75% (v/v) acetonitrile in 10 mM triethylammonium bicarbonate buffer (TEAB). Each fraction was analyzed by liquid chromatography interfaced with tandem mass spectrometry (MS/MS) using a Nano-Acquity HPLC system interfaced with a QExactive HF (Thermo Fisher). A stepped collision energy 32 s/30 s was used for fragmentation. Precursor and fragment ions were analyzed at resolutions 60,000 and 120,000, respectively, and automatic gain control target values at 1e5 with 200 ms maximum injection time and 3e5 with 50 ms maximum injection time, respectively. Data was searched against *Mus musculus* RefSeq v.69 (released January 7, 2015) using the Mascot (Matrix Science) search engine v.2.5 (77989 and 62695 entries actually searched, respectively) running through Proteome Discoverer v.1.4 (DBVersion: 79) (Thermo Fisher) with one or two missed cleavages allowed, respectively; fixed modifications: methylthio (C), TMT6plex (N-term); variable modifications: oxidation (M), deamidation (NQ), and TMT6plex (K); and a tolerance of 10 ppm MS and 0.018 or 0.03 Da MS2, respectively.

For statistical analysis, only spectra with an false discovery rate (FDR) less than 1% (based on a concatenated decoy database) in which all reporter ions were detected were included for downstream analyses. Spectra with isolation interference greater than 30% were excluded as well. Within each TMT experiment, relative protein abundances were quantified by a robust median sweep algorithm (57, 58). Briefly, reporter ion intensities were log2-transformed, spectrum medians of the log2-transformed reporter ion intensities were subtracted (median-polishing), and all reporter ion intensities that belong to the same protein were used as the measure (median) of that protein abundance in the sample. In a final step, the channel medians across all proteins were subtracted to correct for potential loading differences. Statistical inference between two groups of interest was assessed by moderated *t* test statistics (58, 59). For multiple comparison correction, q-values (60) were calculated from the observed *p*-values to control the FDR. That is, if a protein has a q-value of 0.05, we expect to see 5% among the proteins that show smaller *p*-values to be false-positives. Proteins with calculated q-values smaller than 0.05 between different groups can be declared statistically significant. Only proteins quantified by reporter ion spectra in both biological replicates were included for statistical downstream analyses. Normalized protein abundance values for technical replicates were averaged prior to statistical testing.

For proteomics on AAV-infected liver, the samples were digested with the protease trypsin, which has specificity for the amino acids lysine and arginine at the C-terminus of a cleaved peptide. Next, the samples were labeled with unique TMT 10-plex reagent (Thermo Fisher) according to the manufacturer’s protocol. The combined labeled samples were cleaned up from excess TMT tag with a detergent removal spin column (Pierce). The labeled samples were resuspended in an aqueous 5% (v/v) methanol and 0.5% (v/v) formic acid solution and then desalted *via* Oasis HLB cartridges (Waters). Briefly, cartridges were conditioned with 100% (v/v) methanol and equilibrated with 100% (v/v) water. Samples were loaded and then washed twice with 5% (v/v) methanol in water. Peptides were then eluted with 100% (v/v) methanol and dried *via* vacufuge.

Peptides were then fractionated *via* Agilent 3100 OFFGEL isoelectric focusing gel in a 12-well setup according to the manufacturer’s protocol. Upon completion, fractions were separately desalted with Pierce C18 spin columns (Thermo Fisher) following the provided protocol. Column resin was activated with 50% (v/v) acetonitrile and equilibrated with 5% (v/v) acetonitrile +0.5% (v/v) trifluoroacetic acid solutions. Fractions were loaded onto columns and washed with 5% (v/v) acetonitrile +0.5% (v/v) trifluoroacetic acid solution. Samples were eluted *via* two cycles of addition of 70% (v/v) acetonitrile solution to the columns. All sample fractions were dried separately *via* vacufuge.

Each fraction was then analyzed by liquid chromatography–tandem mass spectrometry (LC-MS/MS) using an nLC-1200 nano-flow liquid chromatography system (Thermo Fisher) interfaced with a Q-Exactive mass spectrometer (Thermo Fisher). Precursor and fragment ions were analyzed *via* full scan (resolution of 70,000, automatic gain control target of 3e6, and 40 ms maximum injection time) and a data-dependent MS^2^ top 10 scan (resolution of 17,500, automatic gain control target of 5e4, 150 ms maximum injection time, 0.8 m/z isolation window, 10 s dynamic exclusion period, and normalized collision energy of 27), respectively.

Data from both LC-MS/MS runs for the quantification of Flag-Pgrmc1 and Y113F Flag-Pgrmc1 binding partners and the quantification of the membrane proteome of AAV8 infected *Pgrmc1* KO liver were searched together against the *M. musculus* 10090 Uniprot reference proteome (download date: August 26, 2015) using the Sequest HT search engine running through Proteome Discoverer v.2.1 (Thermo Fisher) with one or two missed cleavages allowed and a precursor mass tolerance of 8 ppm and fragment mass tolerance of 0.02 Da. The trypsin protease cleavage sites permitted were only on the C-terminal sides of lysine and arginine. Dynamic modifications of methionine oxidation and deamidation of asparagine and glutamine, along with static modification of carbamidomethylation of cysteine, were permitted. Peptide assignments were validated using the Target Decoy Peptide Spectral Match Validator node with a relaxed FDR of <0.05 and a strict FDR of <0.01. Peptides from the LC-MS/MS run for the quantification of Flag-Pgrmc1 and Y113F Flag-Pgrmc1 binding partners had a Spectrum File beginning with “03,” while peptides from the LC-MS/MS run for the quantification of the membrane proteome of AAV8-infected *Pgrmc1* KO liver had a Spectrum File beginning with “04.” Included peptides had (1) a Master Accession number, (2) were marked “Unique,” (3) had an isolation interference that was ≤30%, and (4) the Peptide Quan Info was used.

For candidate Flag-Pgrmc1 and Y113F Flag-Pgrmc1 binding partner identification, reporter ion abundances for each spectrum of a protein were summed, then the protein abundance medians for each condition were taken. For Flag-Pgrmc1 and Y113F Flag-Pgrmc1, the ratio of protein abundance to GFP was compared for enrichment. Candidate Flag-Pgrmc1 or Y113F Flag-Pgrmc1 binding partners were those proteins with a fold change (Flag-Pgrmc1/GFP or Y113F Flag-Pgrmc1/GFP) ≥20%. For stringent quantitative analysis of Flag-Pgrmc1 and Y113F Flag-Pgrmc1 binding partners, only peptides present in at least two biological replicates were used to calculate the protein abundance. Peptides present only in Y113F Flag-Pgrmc1 samples were also eliminated.

For quantitative analysis of the membrane proteome of AAV8-infected *Pgrmc1* KO liver, all reporter ion intensities that belong to the same protein were used as the measure (sum) of that protein abundance in the sample. Relative protein abundances were quantified by a robust median sweep algorithm as described above except the measure of a protein’s abundance in a sample was the sum of the peptides. For statistical analysis, conditions were analyzed in R by linear modeling with limma and *p*-values adjusted by the Benjamini and Hochberg FDR method (61).

### GO Term analysis

GO Term Analysis was conducted using PANTHER version 14 (Protein Analysis through Evolutionary Relationships, http://pantherdb.org) (62). A Fisher’s exact test followed by Bonferroni correction was used to identify enriched GO Terms with statistical significance (*p*-value ≤ 0.05). For RNA, up- and downregulated genes were considered to be those genes with fold change ≥40% and a probability of differential expression ≥0.95. Genes were searched by Ref-seq gene name. For protein, proteins were searched by Uniprot ID. In the experiment comparing protein expression in WT and *Pgrmc1* KO liver, differentially expressed proteins were considered to be those proteins with fold change ≥20% and the absolute value of the signal-to-noise ratio ≥2. The differentially expressed proteins were compared with the reference list of all proteins measured in the experiment. In the experiment comparing protein expression in AAV8 GFP, Flag-Pgrmc1, or Y113F Flag-Pgrmc1 infected *Pgrmc1* KO livers, differentially expressed proteins were those with unadjusted *p*-values ≤ 0.05. The differentially expressed proteins were compared with the reference list of all proteins measured in the experiment.

### Total RNA preparation and RNA-seq

Total RNA was prepared from ∼30 mg of snap frozen liver with RNA STAT-60 (Amsbio) reagent according to the manufacturer’s instructions. DNase digestion was performed on-column with an RNAeasy Kit (Qiagen) according to manufacturer’s instructions. RNA concentration was estimated with a NanoDrop (Thermo Fisher). For RNA-seq, equal masses of RNA (2.5 μg) from mice of the same genotype were pooled to create one sample per genotype. Library preparation was completed with an Illumina TruSeq Stranded Total RNA kit. Samples were analyzed on a HiSeq 2500 (Illumina) machine in Rapid Run mode with paired-end 100 bp × 100 bp sequencing. CASAVA 1.8.2 (Illumina) was used to convert BCL files to FASTQ files. Default parameters were used. Rsem-1.2.09 was used for running the alignments as well as generating gene and transcript expression levels. The “rsem-calculate-expression” module was used with the following options: “bowtie-chunkmbs 200,” “calc-ci,” “output-genome-bam,” “paired-end,” and “forward-prob.” The data were aligned to the *M. musculus* mm10 reference genome. The “rsem-run-ebseq” and “rsem-control-fdr” scripts provided by Rsem were used to run EBSeq to perform differential expression analysis. All default parameters were used, except “FDR_rate” was set to 0.05.

### 7-Ethoxycoumarin O-deethylation assay

The 7-ethoxycoumarin O-deethylation (ECOD) assay was conducted as described previously (63). For each genotype, equal amounts of membrane protein from five mice were pooled. Each reaction contained 0.2 mg/ml protein. The substrate 7-ethoxycoumarin was from Sigma. Samples were preincubated at 37 °C for 5 to 10 min. Each reaction was initiated by the addition of NADPH tetrasodium salt (Sigma) to 1 mM and incubated for 30 min at 37 °C while shaking. Control reactions without addition of NADPH were run in parallel. Reactions were quenched upon addition of ice-cold HCl to 0.2 M. After extraction of 7-hydroxycoumarin, 100 μl per sample was transferred to a 96-well plate in triplicate for fluorescence measurement on a FLUOStar Omega plate reader (BMG Labtech) with a 355/460 filter. The fluorescence of each sample was compared with a 7-hydroxycoumarin (Sigma) standard curve including 0, 0.1, 0.5, 1, and 2 pmol standards. The amount of NADPH-dependent product formed was calculated by subtracting the value of the reaction without NADPH from the value of the reaction with NADPH. The NADPH-dependent product formation was used to calculate the reaction velocity normalized to protein amount. Three technical replicates were conducted. Nonlinear regression was carried out on these averaged data and the *K*_m_ and *V*_max_ determined using GraphPad Prism 7.05.

### Caffeine metabolism assay

Pooled membrane protein as described for the ECOD assay was preincubated at 2 mg/ml in 100 mM potassium phosphate, pH 7.4 with an NADPH regenerating system (Corning) at 37 °C for 5 min. Reactions were initiated *via* the addition of caffeine (Sigma) to 50 μM and incubated for 60 min at 37 °C while shaking. Reactions were quenched *via* protein precipitation by direct addition of 50 μl of ice-cold acetonitrile (to 50% v/v) and incubated on ice for 10 min. Precipitate was pelleted by centrifugation for 10 min at 10,000*g* at 4 °C. The supernatant was transferred and dried in a vacuum centrifuge. Samples were reconstituted in 25 μl water prior to mass spectrometry.

Reconstituted samples were resolved using a Dionex Ultimate 3000 uHPLC system and analytes were detected using a coupled Q-Exactive benchtop Orbitrap mass spectrometer (Thermo Fisher). Separation of analytes on an Agilent Polaris C_18_ column (50 × 2.1 mm, 5 μm) was performed using a mobile-phase system of water with 0.1% (v/v) formic acid (mobile phase A) and acetonitrile with 0.1% (v/v) formic acid (mobile phase B) at a flow rate of 400 μl/min. The gradient used was as follows: 0% B from 0 to 4 min, 0 to 5% B from 4 to 15 min, 5 to 100% B from 15 to 16 min, 100% B from 16 to 21 min, 100 to 0% B from 21 to 22 min, and 0% B from 22 to 25 min. Paraxanthine detection was performed in positive ion mode using a transition of m/z 181.0720 > 124.0509. Comparisons of relative amounts of paraxanthine formed were performed using the integrated peak area from the paraxanthine chromatograms.

### p-Nitrophenol hydroxylation assay

The Cyp2e1 activity assay was performed according to Chang, *et al.* (33). For each genotype, equal amounts of membrane protein from four mice were pooled. Briefly, 125 μg of WT or KO liver microsomes was added to ice-cold reaction mix containing 100 μM p-nitrophenol (Sigma, 241326), 1.3 mM NADP+ (Sigma/Roche, 10128031001), 3.3 mM D-glucose-6-phosphate (Sigma, 10127647001), 3.3 mM magnesium chloride (Sigma; M9272), and 0.4 U/ml glucose-6-phosphate dehydrogenase (Worthington, LS003981) in a final volume of 500 μl of 50 mM potassium phosphate buffer, pH 7.4. The reaction was carried out at 37 °C for 1 h, after which the tubes were immediately transferred to ice and 100 μl of TCA was added to stop the reaction. After 5 min incubation on ice, the tubes were centrifuged at 10,000*g* for 5 min at room temperature. In total, 500 μl of the supernatant was added to 300 μl of 2 N NaOH in a fresh tube, mixed, and absorption spectra between 480 nm and 650 nm were recorded using a Genesys 30 (Thermo Fisher) visible spectrophotometer. Peak absorption at 515 nm was used to determine the Cyp2e1 activity using a p-nitrocatechol (Sigma, N15553) standard curve as described in Chang *et al.* (33). Three technical replicates were run; in each of these three runs, three WT and three KO sample replicates were assayed with two no microsome controls. The mean absorbance of the two control samples was subtracted from the absorbances of the three WT and three KO sample replicates within each run.

### Acetaminophen-induced liver injury

Wild-type and *Pgrmc1* KO mice were fasted overnight for 16 h. Mice were then given a single, intraperitoneal injection of 600 mg acetaminophen (Sigma) per kg body weight in 50% saline/DMSO (v/v). All mice were given DietGel Recovery (Clear H_2_O) in the cage. Mice were then euthanized 24 h postinjection. Whole blood was collected *via* cardiocentesis, and serum was separated as described above. The liver was harvested, and the median and left lateral lobes were individually separated. A horizontal, transverse section (including gallbladder) was taken from the median lobe. A diagonal, transverse section to include the hilus was taken from the left lateral lobe. All sections were fixed in 10% neutral buffered formalin (Sigma). Liver sections were processed for histologic analysis as described above.

### Recombinant protein purification and heme loading

Recombinant proteins were expressed in BL21 CodonPlus(DE3)RIPL cells (Agilent) using 200 μM IPTG and overnight shaking at 20 °C. Cells were lysed in B-PER (Thermo Fisher) reagent supplemented with 25 U/ml Benzonase (EMD Biosciences), 2 mM MgCl_2_, 2 mM ATP, 20 mM Imidazole, and 1x protease inhibitors (Protease Complete, Roche) at room temperature for 15 min. The cell lysates were clarified by centrifugation at 80,000*g* at 4 °C for 45 min and purified over a 1 ml HisTrap HP column (GE Healthcare) using a 25 mM-500 mM imidazole gradient in 20 mM HEPES-KOH, pH 7.4; 150 mM NaCl buffer. The purified proteins were desalted using PD10 columns (GE Healthcare) equilibrated with 50 mM HEPES-KOH, pH 7.4; 150 mM NaCl; 2 mM MgCl2; 5% (v/v) glycerol and concentrated using 10,000 MW cutoff filters (Amicon). The N-terminal histidine tag was removed by incubating the concentrated proteins with 50 U/ml Thrombin (GE, 27-0846-01) at room temperature for 16 h, followed by desalting by PD10 column. The recombinant proteins had a residual tetra peptide (Gly-Ser-His-Ser) tag at the N-terminus after thrombin cleavage.

The majority of the bacterially expressed, tag-removed PGRMC1 lacked heme (apo PGRMC1). To test the heme-binding ability of the recombinant PGRMC1 (rPGRMC1), Y113F rPGRMC1, and 3X MUT rPGRMC1, proteins (1 μM) were incubated with 100 μM Hemin (Bovine, Sigma) in PBS for 15 min at 30 °C. Hemin was diluted from a 10 mM stock in DMSO. Thereafter, the proteins were desalted using PBS equilibrated PD10 columns and concentrated using 10,000 MW filters (Amicon). Hemin without any added protein was subjected to the same procedure as a negative control.

### Hemin-binding assay

Hemin was dissolved in DMSO, and the concentration was determined using the extinction coefficient ε_403_ = 170 mM^−1^ cm^−1^ in DMSO (64). During the hemin binding assay, precaution was taken such that DMSO concentration did not exceed 3% (v/v) in the sample. The recombinant PGRMC1 protein (rPGRMC1, Y113F rPGRMC1, or 3X MUT rPGRMC1) (10 μM in 400 μl) was incubated with 0 to 30 μM hemin (2 μM interval) in PBS at room temperature for 16 h, along with the same dilutions of hemin without any added protein. The protein-bound hemin absorbance was measured at 394 nm using the same hemin concentration without any protein as the baseline. The results were fit with a four-parameter log logistic model (Hill Equation) or linear model, using least square method in R (65). From the fitted curves, the K_d_ and B_max_ of hemin binding by PGRMC1 were determined.

### Data analysis

Sample sizes and numbers of biological and technical replicates are noted for each experiment. Statistical tests (*t*-tests and ANOVAs followed by Tukey HSD post-hoc tests) were performed in GraphPad Prism versions 7.05 to 8.2.0. Analysis of large datasets was performed using R.

## Data availability

RNA-Seq data are deposited at NCBI GEO (https://www.ncbi.nlm.nih.gov/geo/), accession GSE174375. The mass spectrometry proteomics data have been deposited to the ProteomeXchange Consortium *via* the PRIDE (66) partner repository (http://www.ebi.ac.uk/pride) with the dataset identifiers PXD028238, PXD028284, and PXD028288. All remaining data are contained within the article and supporting information.

## Supporting information

This article contains supporting information.
