# Supplementary material for: Progesterone receptor membrane component 1 (PGRMC1) binds and stabilizes cytochromes P450 through a heme-independent mechanism
Source: J Biol Chem. 2021 Oct 20;297(5):101316. doi: 10.1016/j.jbc.2021.101316 (PMC8591507; doi:10.1016/j.jbc.2021.101316)
Supplement: Figures S1–S6 [file mmc1.pdf]

**Supplemental Information**

**TITLE:** Progesterone receptor membrane component 1 (PGRMC1) binds and stabilizes cytochromes P450 through a heme-independent mechanism

**AUTHORS:** Meredith R. McGuire, Debaditya Mukhopadhyay, Stephanie L. Myers, Eric P. Mosher, Rita T. Brookheart, Kai Kammers, Alfica Sehgal, Ebru S. Selen, Michael J. Wolfgang, Namandjé N. Bumpus, and Peter J. Espenshade

**Table of Contents**


| Contents | Page or File Name |
|---|---|
| Title and Author | S-1 |
| Table of Contents | S-2 |
| Figure S1 | S-3 |
| Figure S2 | S-5 |
| Figure S3 | S-8 |
| Figure S4 | S-10 |
| Figure S5 | S-12 |
| Figure S6 | S-14 |
| Tables S1.1-S1.3: CBC, Clinical Chemistry, and NMR Metabolites for *Pgrmc1* KO mice | mmc2.xlsx |
| Tables S2.1 and S2.2: Binding partners of Flag-Pgrmc1 and Y113F Flag-Pgrmc1 in Mouse Liver | mmc3.xlsx |
| Table S3.1-S3.5: Pgrmc1 KO liver proteomics | mmc4.xlsx |
| Table S4.1-S4.4: Pgrmc1 KO liver RNA-seq | mmc5.xlsx |
| Table S5.1-S5.6: AAV Flag-Pgrmc1 and Y113F Flag-Pgrmc1 liver membrane proteome | mmc6.xlsx |
| Table S6: Stringent quantitative analysis of Flag-Pgrmc1 and Y113F Flag-Pgrmc1 binding partners | mmc7.xlsx |

## Fig. S1

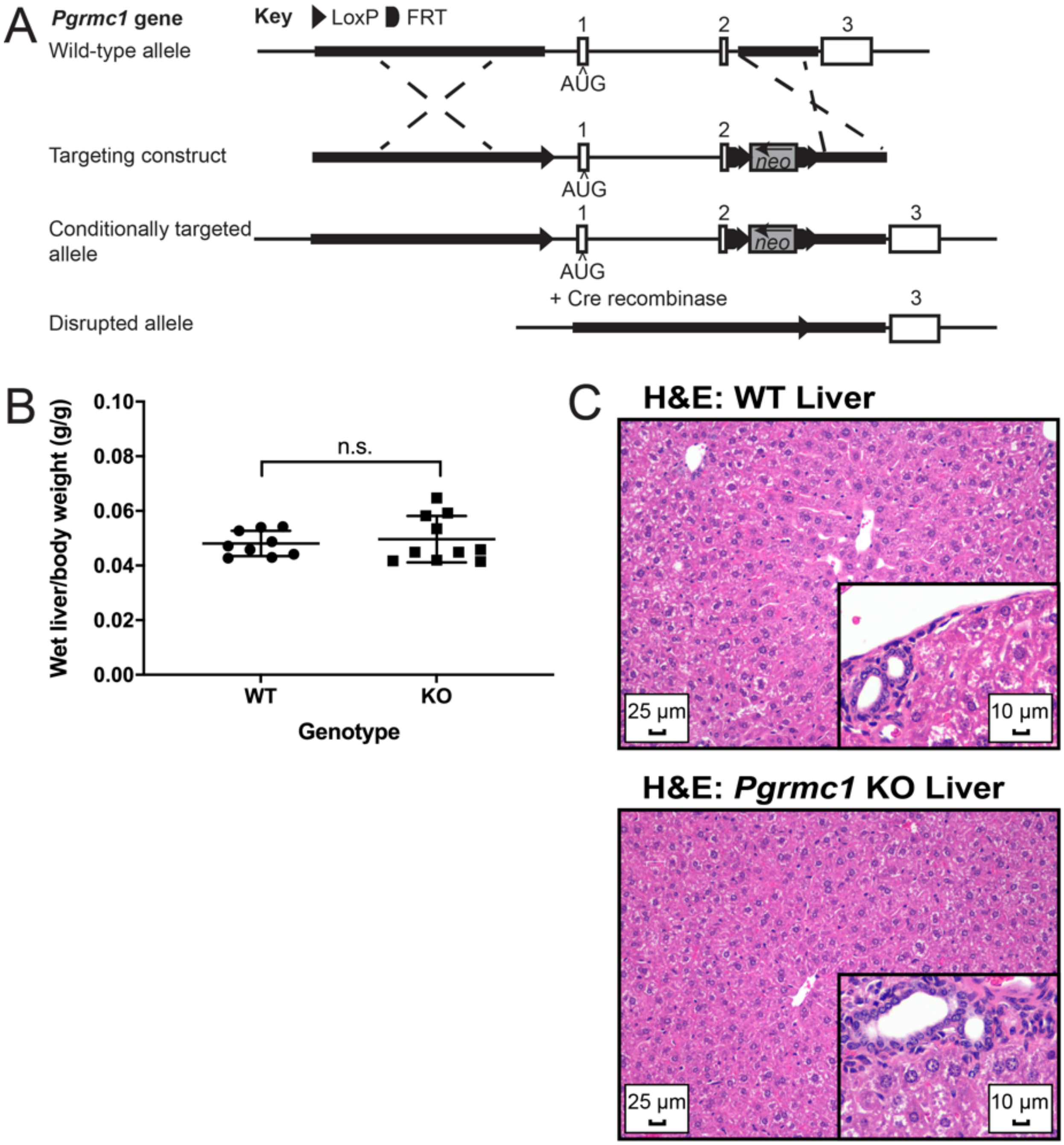
A
Pgrmc1 gene
Wild-type allele
Key
LoxP
FRT
1
2
3
AUG
Targeting construct
neo
Conditionally targeted allele
+ Cre recombinase
Disrupted allele
B
Wet liver/body weight (g/g)
0.10
0.08
0.06
0.04
0.02
0.00
n.s.
WT
KO
Genotype
C
H&E: WT Liver
25 µm
10 µm
H&E: Pgrmc1 KO Liver
25 µm
10 µm

**FIGURE S1: Generation of a *Pgrmc1* knockout mouse**

A) Schematic of the *Pgrmc1* gene-targeting strategy. The map of the *Mus musculus* wildtype allele spans the full *Pgrmc1* gene on the X chromosome. Cre recombinase deletes 4 kb of genomic sequence including exons 1 and 2, which are flanked by LoxP sites. The disrupted allele deletes amino acid residues 1 to 161 of the Pgrmc1 protein, which code for the protein transmembrane domain and the cytochrome b5-like domain. Open rectangles denote exons.

B) Liver weight normalized to body weight for *Pgrmc1* KO mice (WT 0.048 ± 0.0015, KO 0.050 ± 0.0027). Error bars are 1 SD (WT n=9, KO n=10; Student's t-test; n.s. is not significant).

C) Representative H&E stained sections of formalin-fixed liver tissue from *ad libitum* fed *Pgrmc1* KO mice showing the portal vein at 200X magnification (inset shows ducts at 400X magnification).

**Fig. S2**

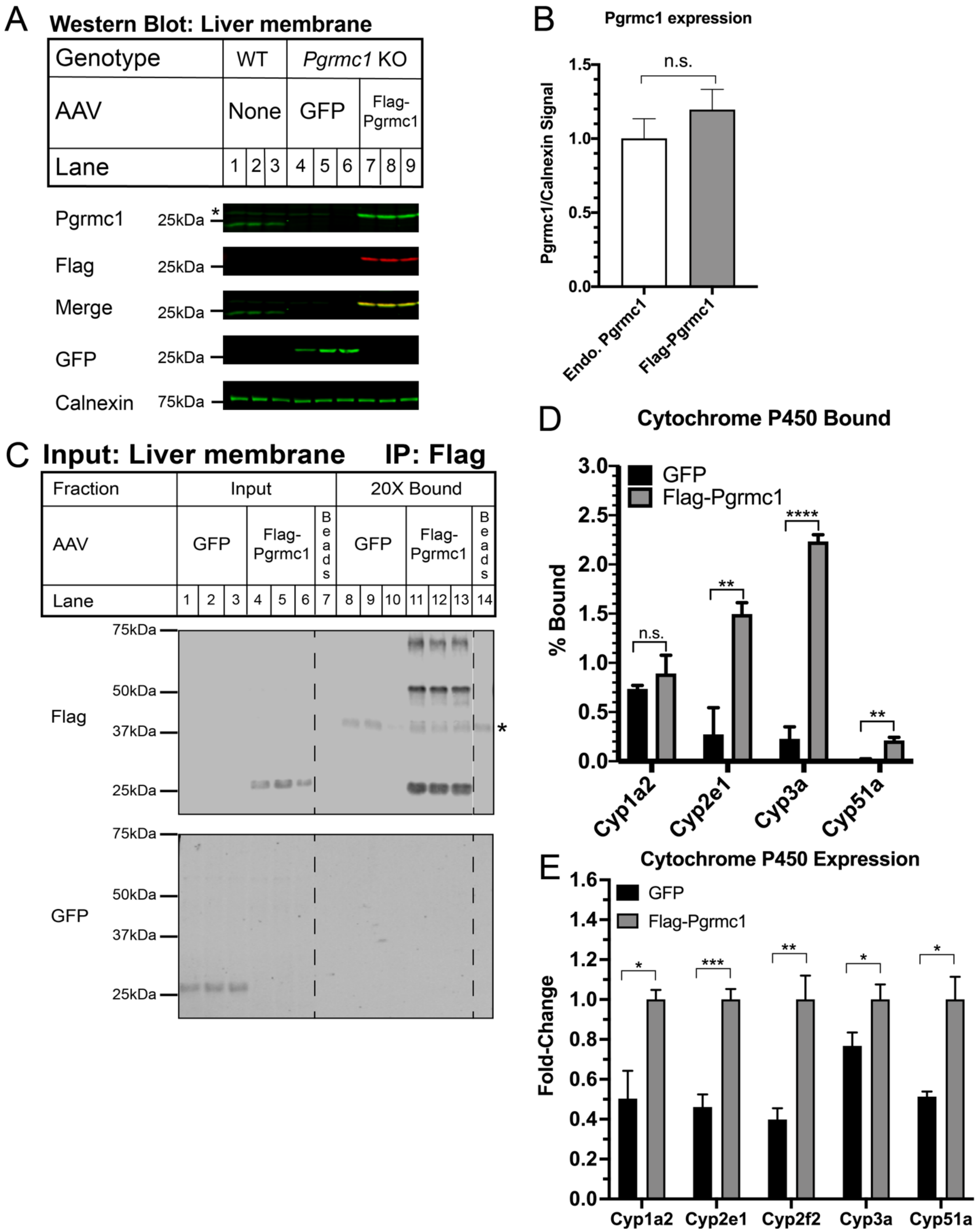
A
Western Blot: Liver membrane
Genotype
WT
Pgrmc1 KO
AAV
None
GFP
Flag-Pgrmc1
Lane
1 2 3 4 5 6 7 8 9
Pgrmc1
Flag
Merge
GFP
Calnexin
25kDa
75kDa
*
B
Pgrmc1 expression
Pgrmc1/Calnexin Signal
n.s.
1.5
1.0
0.5
0.0
Endo. Pgrmc1
Flag-Pgrmc1
C
Input: Liver membrane
IP: Flag
Fraction
Input
20X Bound
AAV
GFP
Flag-Pgrmc1
Beads
Lane
1 2 3 4 5 6 7 8 9 10 11 12 13 14
Flag
GFP
75kDa
50kDa
37kDa
25kDa
*
D
Cytochrome P450 Bound
GFP
Flag-Pgrmc1
% Bound
3.0
2.5
2.0
1.5
1.0
0.5
0.0
n.s.
**
****
**
Cyp1a2
Cyp2e1
Cyp3a
Cyp51a
E
Cytochrome P450 Expression
GFP
Flag-Pgrmc1
Fold-Change
1.6
1.4
1.2
1.0
0.8
0.6
0.4
0.2
0.0
*
***
**
*
*
Cyp1a2
Cyp2e1
Cyp2f2
Cyp3a
Cyp51a

**FIGURE S2: Validation of Flag co-immunoprecipitation from AAV infected mice**

A) Western blot of liver membrane fraction from uninfected WT mice and *Pgrmc1* KO mice infected with AAV8 GFP or AAV8 Flag-Pgrmc1 as in Fig. 1 C. "Merge" is the combination of the "Pgrmc1" and "Flag" panels. Calnexin is a loading control. Each lane is a biological replicate (WT/None n=3, KO/AAV8 GFP n=3, KO/AAV8 Flag-Pgrmc1 n=3). (* denotes a background band)

B) Quantification of Pgrmc1 protein expression from A. Pgrmc1 signal was normalized to the calnexin signal and plotted relative to endogenous (endo) Pgrmc1 protein levels. Error bars are 1 SD. (Endo. Pgrmc1 n=3, Flag-Pgrmc1 n=3; Welch's t-test; n.s. denotes not significant).

C) Representative western blot of one of three technical replicates of Flag co-immunoprecipitation (1X Input, 20X Bound) performed on liver membrane fractions from *Pgrmc1* KO mice infected with AAV8 as in Fig. 1 C, D. Each panel is a montage from a single membrane with dashed lines denoting removed lanes. Each lane is a biological replicate (GFP n=3, Flag-Pgrmc1 n=3) or bead only control. (* denotes IgG)

D) Quantification of cytochromes P450 in the bound fraction of the Flag co-immunoprecipitation for Fig. 1 D. Within each biological replicate, the ratio of cytochrome P450 expression in the Bound fraction to the Input fraction was quantified. Error bars are 1 SD. (GFP n=3, except for Cyp51a where n=2, Flag-Pgrmc1 n=3; Welch's t-test for each cytochrome P450; n.s. denotes not significant, ** $p \leq 0.01$, *** $p \leq 0.0001$, **** $p \leq 0.0001$).

E) Quantification of cytochrome P450 protein in the input of the Flag co-immunoprecipitation for Fig. 1 D. For each condition, biological replicates were averaged, and the average for each condition was normalized to the Flag-Pgrmc1 condition. Error is 1 SD. (GFP n=3, Flag-Pgrmc1 n=3; Welch's t-test for each cytochrome P450; * $p \leq 0.05$, ** $p \leq 0.01$, *** $p \leq 0.001$)

## Fig. S3

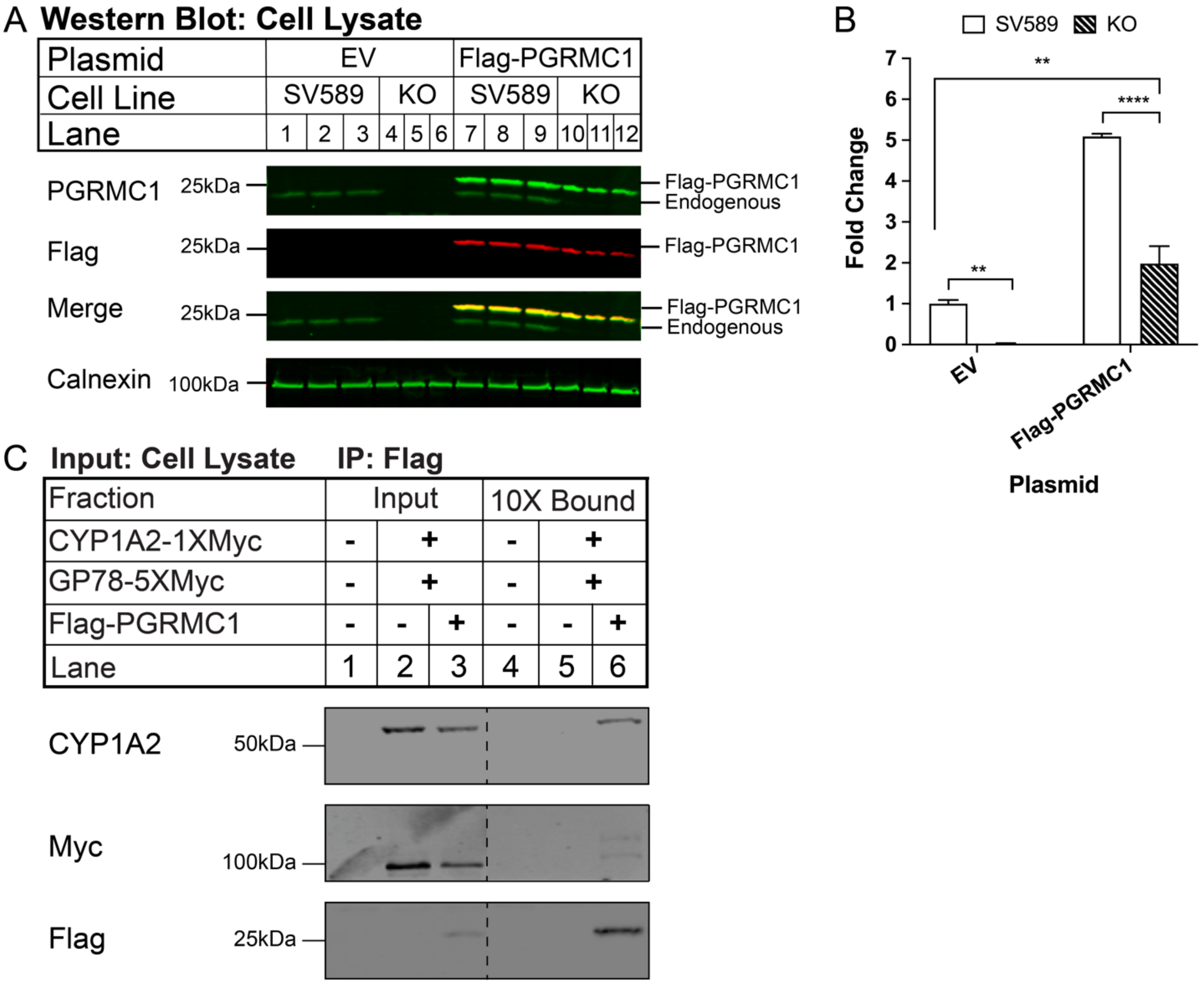
A Western Blot: Cell Lysate
Plasmid
EV
Flag-PGRMC1
Cell Line
SV589
KO
SV589
KO
Lane
1 2 3 4 5 6 7 8 9 10 11 12
PGRMC1
25kDa
Flag-PGRMC1
Endogenous
Flag
25kDa
Flag-PGRMC1
Merge
25kDa
Flag-PGRMC1
Endogenous
Calnexin
100kDa
B
SV589
KO
Fold Change
0 1 2 3 4 5 6 7
**
****
**
EV
Flag-PGRMC1
Plasmid
C Input: Cell Lysate IP: Flag
Fraction
Input
10X Bound
CYP1A2-1XMyc
GP78-5XMyc
Flag-PGRMC1
Lane
1 2 3 4 5 6
CYP1A2
50kDa
Myc
100kDa
Flag
25kDa

**FIGURE S3: PGRMC1 binds CYP1A2 in human SV589 cells**

A) *PGRMC1* KO SV589 cells do not express PGRMC1 protein. *PGRMC1* was knocked out in human fibroblast SV589 cells via CRISPR genome editing. Knockout was confirmed by western blotting cell lysates. Cells were transfected with 10 µg of empty vector (EV) or Flag-PGRMC1 plasmid. For each condition, each lane is a technical triplicate of transfection.

B) Quantification of PGRMC1 expression from A. PGRMC1 signals measured in the region spanning both Flag-tagged and endogenous PGRMC1 were quantified and normalized to the calnexin signal for each lane. Technical triplicates were averaged and normalized to the EV control. Error bars are 1 SD. (n=3 per condition, two-way ANOVA and Tukey's HSD; ** $p \leq 0.01$, **** $p \leq 0.0001$)

C) *PGRMC1* KO cells were co-transfected with CYP1A2-1XMyc (5 µg), GP78-5XMyc (0.05 µg) as a specificity control, and Flag-PGRMC1 (10 µg) plasmids in a 10-cm plate. At 24 hr post-transfection, Flag-PGRMC1 was immunoprecipitated with Flag M2 agarose beads. Bound fractions were eluted in 1X SDS sample buffer without β-mercaptoethanol. Input (1X) and bound (10X) fractions were analyzed by western blotting using an anti-CYP1A2 antibody to detect CYP1A2-1XMyc and anti-c-Myc antibody to detect GP78-5XMyc. Each panel is a montage from a single membrane with dashed lines denoting removed lanes. Panels are representative of 3 biological replicates.

**Fig. S4**

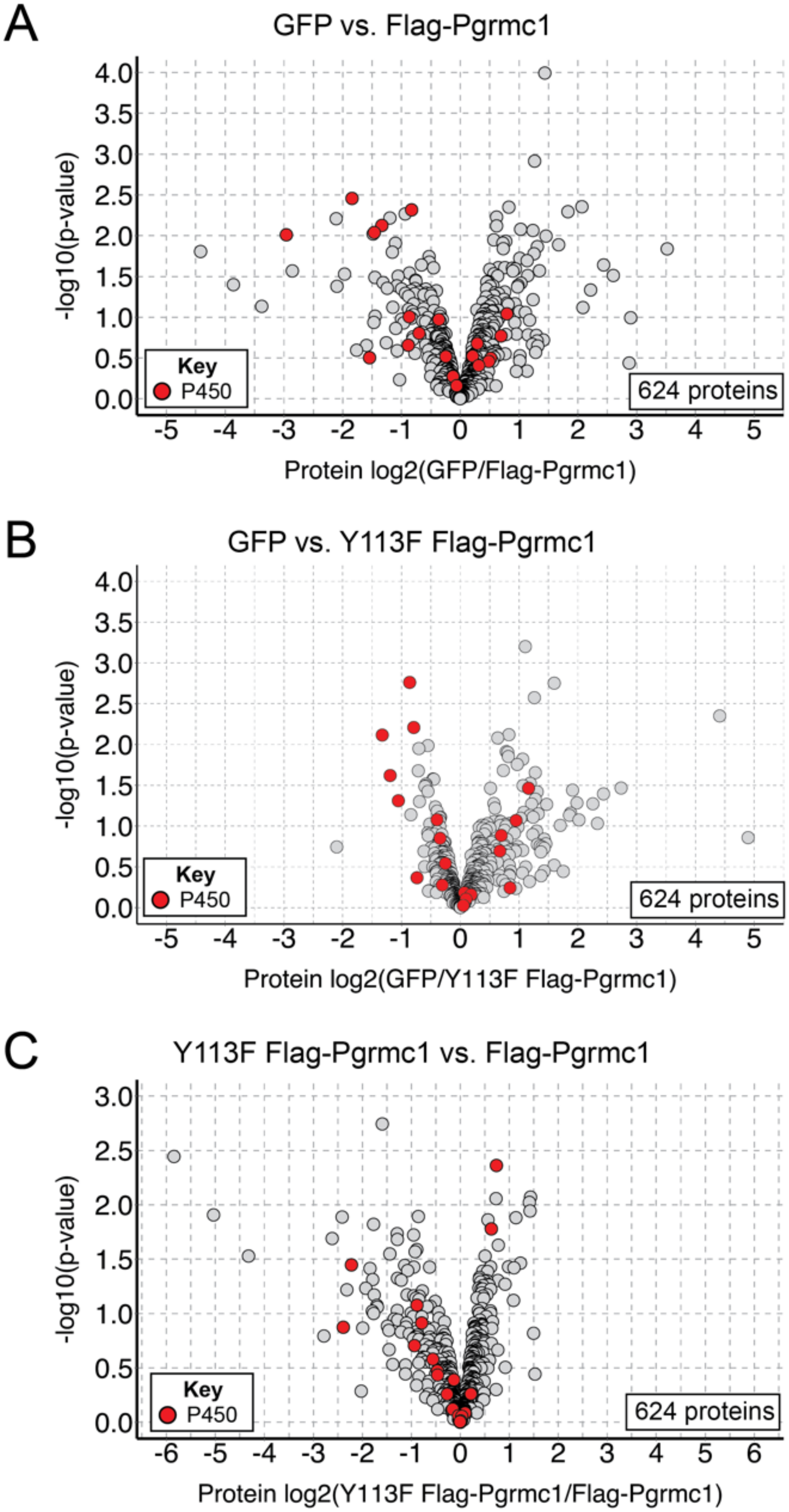
A
GFP vs. Flag-Pgrmc1
-log10(p-value)
4.0
3.5
3.0
2.5
2.0
1.5
1.0
0.5
0.0
-5 -4 -3 -2 -1 0 1 2 3 4 5
Protein log2(GFP/Flag-Pgrmc1)
Key
P450
624 proteins
B
GFP vs. Y113F Flag-Pgrmc1
-log10(p-value)
4.0
3.5
3.0
2.5
2.0
1.5
1.0
0.5
0.0
-5 -4 -3 -2 -1 0 1 2 3 4 5
Protein log2(GFP/Y113F Flag-Pgrmc1)
Key
P450
624 proteins
C
Y113F Flag-Pgrmc1 vs. Flag-Pgrmc1
-log10(p-value)
3.0
2.5
2.0
1.5
1.0
0.5
0.0
-6 -5 -4 -3 -2 -1 0 1 2 3 4 5 6
Protein log2(Y113F Flag-Pgrmc1/Flag-Pgrmc1)
Key
P450
624 proteins

**FIGURE S4: Membrane proteome of Y113F Flag-Pgrmc1-expressing *Pgrmc1* KO mice**

*Pgrmc1* KO mice were infected with 5 x $10^{11}$ particles of AAV8 GFP, AAV8 Flag-Pgrmc1, or AAV8 Y113F Flag-Pgrmc1 and sacrificed after 8 days. Steady state protein levels from liver membrane fractions were quantified by mass spectrometry with isobaric tagging (tmt) with three biological replicates per group. A total of 624 proteins were detected in all three mice per condition. The log2 fold change in protein expression was plotted against the unadjusted p-value. In A and B, GFP condition is compared to the Flag-Pgrmc1 and Y113F Flag-Pgrmc1 conditions, respectively. In C, the Y113F Flag-Pgrmc1 and Flag-Pgrmc1 conditions are compared to each other. Cytochromes P450 are colored red.

# Fig. S5

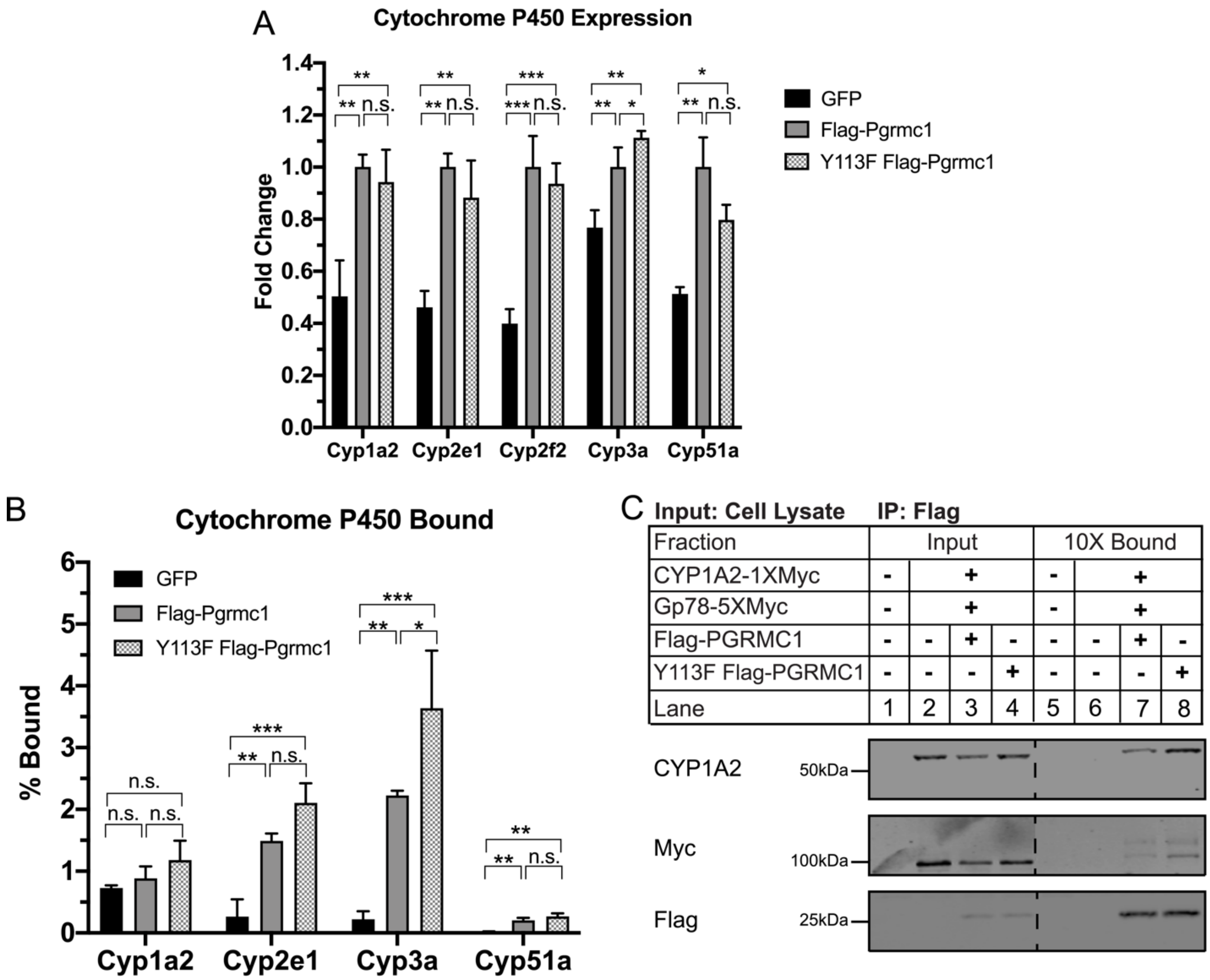
A
Cytochrome P450 Expression
Fold Change
0.0
0.2
0.4
0.6
0.8
1.0
1.2
1.4
Cyp1a2
Cyp2e1
Cyp2f2
Cyp3a
Cyp51a
GFP
Flag-Pgrmc1
Y113F Flag-Pgrmc1
** n.s.
***
*
B
Cytochrome P450 Bound
% Bound
0
1
2
3
4
5
6
GFP
Flag-Pgrmc1
Y113F Flag-Pgrmc1
Cyp1a2
Cyp2e1
Cyp3a
Cyp51a
n.s.
**
***
*
C
Input: Cell Lysate IP: Flag
Fraction Input 10X Bound
CYP1A2-1XMyc - + - +
Gp78-5XMyc - + - +
Flag-PGRMC1 - - + - - - + -
Y113F Flag-PGRMC1 - - - + - - - +
Lane 1 2 3 4 5 6 7 8
CYP1A2
50kDa
Myc
100kDa
Flag
25kDa

**FIGURE S5: Y113F PGRMC1 binds and stabilizes cytochromes P450**

A) Quantification of Input fraction signal from Fig. 5B. Error is 1 SD (n=3 biological replicates per condition, except for Cyp51a in which GFP was n=2; one-way ANOVA and Tukey HSD for each P450; n.s. denotes not significant, * $p \leq 0.05$, ** $p \leq 0.01$, ***$p \leq 0.001$)

B) Quantification of cytochromes P450 in the bound fraction of the Flag co-immunoprecipitation for Fig. 5 B. Within each biological replicate, the ratio of cytochrome P450 expression in the Bound fraction to the Input fraction was quantified. Error is 1 SD (GFP n=3 except Cyp51a where n=2, Flag-Pgrmc1 n=3, Y113F Flag-Pgrmc1 n=3; one-way ANOVA and Tukey HSD for each P450; n.s. denotes not significant, * $p \leq 0.05$, ** $p \leq 0.01$, ***$p \leq 0.001$)

C) Y113F Flag-PGRMC1 binds CYP1A2 in human SV589 cells. *PGRMC1* KO cells were co-transfected with CYP1A2-1XMyc (5 µg), GP78-5XMyc (0.05 µg) as a specificity control, and Flag-PGRMC1 (10 µg) or Y113F Flag-PGRMC1 (10 µg) plasmids in a 10-cm plate. At 24 hr post-transfection, Flag-PGRMC1 was immunoprecipitated with Flag M2 agarose beads. Bound fractions were eluted in 1X SDS sample buffer without β-mercaptoethanol. Input (1X) and bound (10X) fractions were analyzed by western blotting using an anti-CYP1A2 antibody to detect CYP1A2-1XMyc and anti-c-Myc antibody to detect GP78-5XMyc. Each panel is a montage from a single membrane with dashed lines denoting removed lanes. Panels are representative of 3 biological replicates. Lanes 1-3 and 5-7 are the same images as Fig. S3 C Lanes 1-3 and 4-6.

# Fig. S6

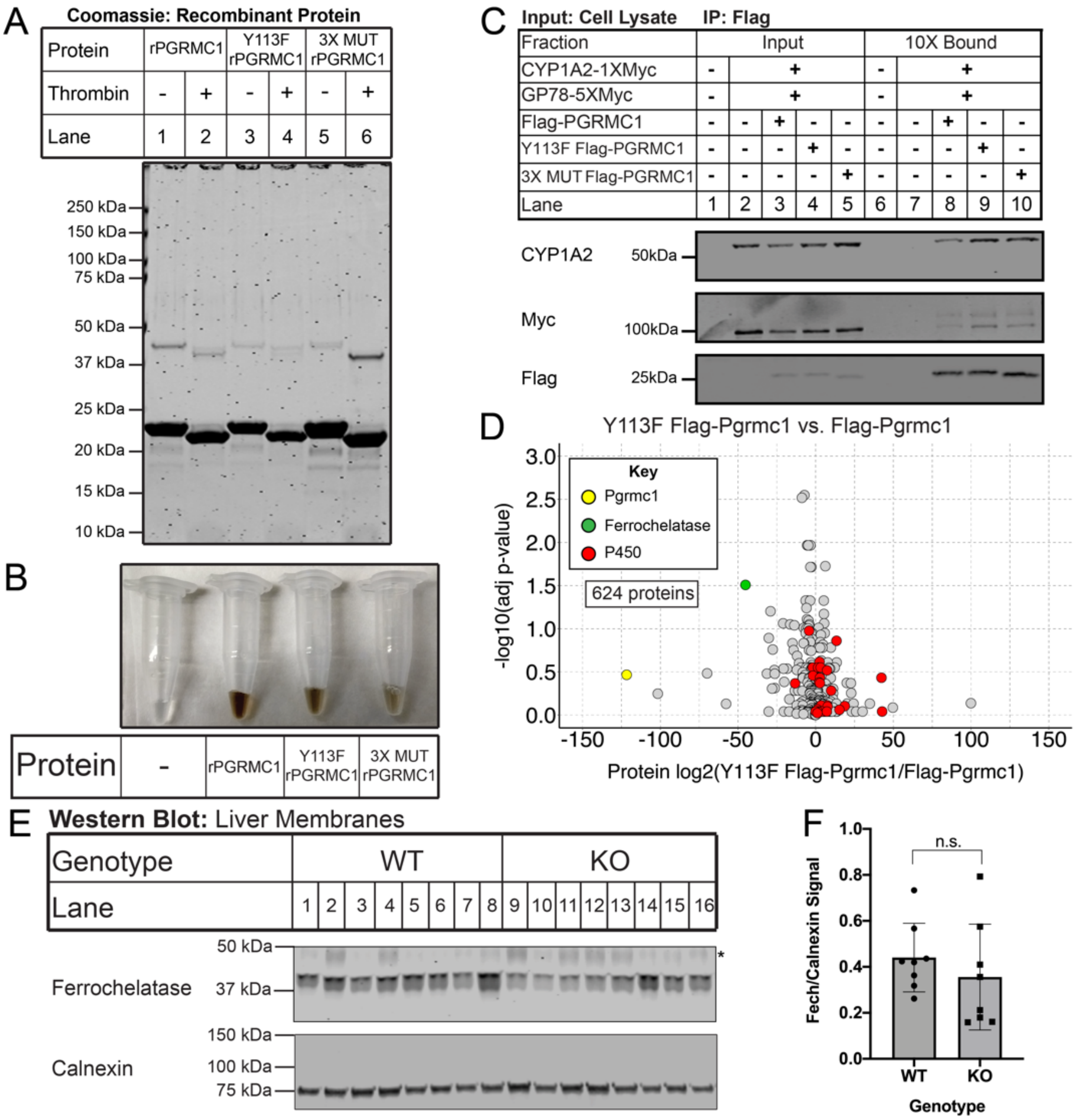
A
Coomassie: Recombinant Protein
Protein
rPGRMC1
Y113F rPGRMC1
3X MUT rPGRMC1
Thrombin
Lane
250 kDa
150 kDa
100 kDa
75 kDa
50 kDa
37 kDa
25 kDa
20 kDa
15 kDa
10 kDa
B
Protein
-
rPGRMC1
Y113F rPGRMC1
3X MUT rPGRMC1
C
Input: Cell Lysate IP: Flag
Fraction
Input
10X Bound
CYP1A2-1XMyc
GP78-5XMyc
Flag-PGRMC1
Y113F Flag-PGRMC1
3X MUT Flag-PGRMC1
Lane
CYP1A2
50kDa
Myc
100kDa
Flag
25kDa
D
Y113F Flag-Pgrmc1 vs. Flag-Pgrmc1
Key
Pgrmc1
Ferrochelatase
P450
624 proteins
-log10(adj p-value)
Protein log2(Y113F Flag-Pgrmc1/Flag-Pgrmc1)
E
Western Blot: Liver Membranes
Genotype
WT
KO
Lane
Ferrochelatase
Calnexin
F
n.s.
Fech/Calnexin Signal
Genotype
WT
KO

**FIGURE S6: Pgrmc1 binds cytochromes P450 in a heme-independent manner, while binding to ferrochelatase is sensitive to the Y113F mutation in PGRMC1.**

A) Coomassie brilliant blue-stained gel of recombinant purified truncated (a.a. 43-195) rPGRMC1, Y113F rPGRMC1 and Y113F, K163A, K164A (3X MUT) rPGRMC1. 6X His tagged proteins were expressed in *Escherichia coli* and purified by FPLC over a HisTrap HP column with a 25 mM-500 mM imidazole gradient. Proteins were desalted using PD10 columns and concentrated using 10,000 MW cutoff filters into HEPES buffer. The N-terminal histidine tag was removed by incubating the concentrated proteins with 50 U/ml Thrombin at room temperature for 16 hr, followed by desalting using a PD10 column.

B) Image of purified rPGRMC1, Y113F rPGRMC1 and Y113F, 3X MUT rPGRMC1 proteins in solution. Proteins (1 μM) were incubated with 100 μM Hemin in PBS for 15 min at 30°C. Hemin without any added protein was subjected to the same procedure as a negative control. The deep reddish-brown color of rPGRMC1 and Y113F rPGRMC1 is characteristic of heme-binding proteins.

C) *PGRMC1* KO cells were co-transfected with CYP1A2-1XMyc (5 μg), GP 78-5XMyc (0.05 μg) as a specificity control, and Flag-PGRMC1, Y113F Flag-PGRMC1, or 3X MUT Flag-PGRMC1 (10 μg) plasmids in a 10-cm plate. At 24 hr post-transfection, Flag-PGRMC1 was immunoprecipitated with Flag M2 agarose beads. Bound fractions were eluted in 1X SDS sample buffer without β-mercaptoethanol. Input (1X) and bound (10X) fractions were subjected to western blotting using an anti-CYP1A2 antibody to detect CYP1A2-1XMyc and anti-c-Myc antibody to detect GP78-5XMyc. Panels are representative of 3 biological replicates. Lanes 1-4 and 6-9 are the same images as Fig. S5 C Lanes 1-4 and 5-8.

D) Stringent quantitative analysis (limma) comparing Y113F Flag-Pgrmc1 and Flag-Pgrmc1 binding partners in AAV infected *Pgrmc1* KO liver. Samples were prepared and binding partners identified by mass spectrometry as in Fig. 1 C. The log2 fold change [log2(Y113F Flag-Pgrmc1/Flag-Pgrmc1)] in abundance was plotted against the log transformed FDR-adjusted p-value [-log10(adj p-value)]. Cytochromes P450 are colored red, Pgrmc1 is colored yellow, and ferrochelatase is colored green.

E) Western blots of liver membrane fractions for ferrochelatase. The samples used for immunoblotting are the same as in Fig. 2B. The calnexin loading control blot has been duplicated here for convenience. Each lane is a biological replicate (WT n=8, KO n=8). (* denotes a background band)

F) Quantification of ferrochelatase expression from F. Ferrochelatase signal intensity for each lane was normalized to calnexin. Error bars are 1 SD. (WT n=8, KO n=8; n.s. is not significant).
